# Risk prediction models for contrast-induced acute kidney injury in patients with acute coronary syndromes: a systematic review and meta-analysis

**DOI:** 10.3389/fmed.2025.1629369

**Published:** 2025-09-18

**Authors:** Lu Zhang, Xuehua Cao, Yanmei Yang, Songying Fu, Yu Jia, Wanqing Hu, Feng Xiang

**Affiliations:** ^1^School of Nursing, Chengdu University of Traditional Chinese Medicine, Sichuan, China; ^2^Department of Gynecology Nursing, Sichuan Provincial People’s Hospital, University of Electronic Science and Technology of China, Sichuan, China

**Keywords:** acute coronary syndromes, percutaneous coronary intervention, coronary angiography, contrast-induced acute kidney injury, prediction model, meta-analysis

## Abstract

**Background:**

Percutaneous coronary intervention (PCI) has become a crucial method for the treatment of acute coronary syndromes (ACS), which includes ST-segment elevation myocardial infarction (STEMI), non-ST-segment elevation myocardial infarction (NSTEMI), and unstable angina (UA). However, contrast-induced acute kidney injury(CI-AKI) is one of its serious complications. A growing number of models have been used to predict ACS patients undergoing coronary angiography (CAG) or PCI, but the predictive efficacy of these models is unclear.

**Methods:**

We systematically searched PubMed, Web of Science, The Cochrane Library, and Embase from the inception to May 18, 2024. This study excluded non-English studies to reduce potential language bias. The Prediction Model Risk of Bias Assessment Tool (PROBAST) was used to evaluate bias risk and applicability of the studies in the prediction model, and the area under the curve (AUC) values of the models were meta-analyzed by Stata 15.0 software.

**Results:**

13,834 articles were retrieved, and 16 studies were finally included after screening. The incidence of CI-AKI in patients with ACS underwent PCI or CAG ranged from 4.66 to 19.85%. The developed models exhibited a pooled AUC of 0.804 (95% CI: 0.772–0.836), while the validation models demonstrated a pooled AUC of 0.785 (95% CI: 0.747–0.823). However, significant heterogeneity was observed in both the development and validation cohorts (89.7 and 84.8%, respectively), along with publication bias (*p* < 0.05). All included studies were assessed as having a high risk of bias, mainly due to inappropriate data sources and bias in statistical analysis.

**Conclusion:**

No existing model for CI-AKI after CAG or PCI can currently be recommended for routine use due to the high risk of bias and the lack of external validation. Researchers should follow PROBAST and use a prospective design with a large sample size to improve the quality of prediction models and provide better clinical value.

**Systematic review registration:**

https://www.crd.york.ac.uk/PROSPERO/view/CRD42024573128.

## 1 Introduction

Globally, it is Acute coronary syndromes(ACS) has estimated that more than 7 million people are diagnosed with ACS each year (1). It constitutes a major public health challenge, characterized by high morbidity and mortality, and encompasses three types: ST-segment elevation myocardial infarction (STEMI), non-ST-segment elevation myocardial infarction (NSTEMI), and unstable angina (UA) (2, 3). With the advancement in current diagnostic and therapeutic technology, coronary angiography(CAG) and percutaneous coronary intervention (PCI) have emerged as pivotal methods for treating ACS, while simultaneously serving as the key strategies to decrease mortality rates. Both procedures necessitate the administration of contrast agents: the former serves to visualize coronary artery lesions, and the latter entails interventional therapy for said lesions. However, a serious complication known as contrast-induced acute kidney injury (CI-AKI) may occur following CAG or PCI. CI-AKI is the third leading cause of hospital-acquired renal insufficiency (4). Some studies have shown that the incidence of CI-AKI ranges from 5.1 to 10.5% in patients who underwent CAG or PCI (5, 6).

Acute myocardial infarction (AMI), including STEMI and NSTEMI, as the most severe type of ACS, requires particular attention regarding subsequent risk of CI-AKI. This is not only due to hemodynamic instability but also because the systemic inflammatory response triggered by AMI and the unique clinical phenotypes of patients collectively create a high-risk internal environment prone to kidney injury (7, 8).

CI-AKI is associated with an increased risk of mortality, cardiovascular events, hemodialysis, renal failure, and prolonged hospitalization (9, 10). This imposes a heavy economic burden on patients and society. Brinjikji et al.’s (11) and Tian et al.’s (12) studies conducted systematic reviews and meta-analyses on CI-AKI, but did not focus on ACS patients and were also unrelated to the predictive models. Both the Mehran score (13) and the score (14) based on the data from the National Cardiovascular Data Registry Cath-PCI registry were validated to have good predictive performance, but neither of them can be used to identify high-risk patients (such as patients with chronic renal insufficiency). Subsequently, some researchers have further developed prediction models. However, the majority of current prediction models are designed for subtypes of ACS, such as STEMI or NSTEMI, while prediction models that focus on ACS as a whole are relatively rare. Although David et al. (15) conducted a systematic review and meta-analysis of the prediction models for CI-AKI, they not only failed to focus on patients with ACS but also did not apply Prediction Model Risk of Bias Assessment Tool (PROBAST), which was developed in 2019 and is now widely used. Moreover, the current CI-AKI prediction models lack of methodological standardization in model construction and validation.

Therefore, this study aimes to screen and systematically review existing CI-AKI risk prediction models developed and published for patients with ACS, and conduct a meta-analysis. It will provide a valuable reference for clinical application and future research.

## 2 Manuscript formatting

### 2.1 Methods

This study followed the guidelines for Preferred Reporting Items for Systematic reviews and Meta-Analyses (PRISMA). The Prediction Model Risk of Bias Assessment Tool(PROBAST) was used to assess the risk of bias in the mode. The study protocol was registered on PROSPERO (registration number: CRD42024573128).

#### 2.1.1 Search strategy

We searched the English databases, including PubMed, Web of Science, The Cochrane Library and Embase, which were searched from the inception of the databases until May 18, 2024. The search strategy combined the use of MeSH terms and free words, with the words connected by the logical operators”AND” and “OR.” The keywords including: “acute coronary syndrome,” “percutaneous coronary intervention,” “acute kidney injury,” “contrast-induced acute kidney injury,” “predictive model*,” “risk factor*,” and so on. Detailed strategies for searching can be found in the Supplementary materials.

We utilized the PICOTS system, recommended by the Critical Appraisal and Data Extraction for Systematic Reviews of Prediction Modeling Studies (CHARMS) checklist for the systematic review. The key items of our systematic review are described as follows:

P (Population): Patients with ACS and underwent PCI or CAG.
I (Intervention model): Developed and published risk prediction models for CI-AKI in patients with ACS (predictors ≥ 2).
C (Comparator): No competing model.
O (Outcome): The findings focused on CI-AKI, rather than its subgroups.
T (Timing): The outcome was predicted after evaluating basic information at admission, clinical scoring scale results, and laboratory indicators.
S (Setting): The purpose of the risk prediction model is to predict the occurrence of CI-AKI after PCI or CAG in patients with ACS, to facilitate the implementation of preventive measures and prevent the occurrence of adverse events.

#### 2.1.2 Inclusion and exclusion criteria

The inclusion criteria for studies were: (1) studies involving patients with ACS (including STEMI, NSTEMI and UA) and underwent PCI or CAG; (2) an observational study design; (3) reported a prediction model; (4) the outcome of interest was CI-AKI.

The exclusion criteria were: (1) studies that did not develop a predictive model; (2) outcome of CI-AKI appeared in subgroups; (3) non-English studies were excluded to avoid translation bias and ensure accurate information extraction; (4) the full text could not be retrieved; (5) conference abstracts, letters, gray literature; (6) unable to extract data.

#### 2.1.3 Study selection and screening

The selection process for the study was conducted independently by two researchers. Firstly, duplicate studies, conference abstracts and letters were removed. Secondly, the remained studies were assessed based on their titles and abstracts to determine their eligibility. Then the full text of the inclusion and exclusion criteria was reviewed after they were applied. Finally, studies with inconsistent patients and outcomes were removed, and 16 studies were included. When there are differences in the screening results, the three researchers will discuss and negotiate together to reach a consensus.

#### 2.1.4 Data extraction

Two researchers independently screened the search results, extracted data and cross-checked the results. Disagreements were resolved through discussion with a third researcher.

The information extracted from the remained studies was categorized into two parts: (1) Basic information: author, title, year of publication, research design, participants, interventional procedure type, data source, study period, hydration protocol, contrast protocol, country, sample size and cases. (2) model information: missing data handling, variable selection method, model development method, calibration method, validation method, final predictors, model performance (If more than one model was established, the optimal AUC was extracted.), model presentation and clinical application. The above information was extracted by one person and checked by another person to ensure the accuracy and consistency of the results.

#### 2.1.5 Quality assessment

To assess the potential risk of bias in the included predictive model studies, the Prediction Model Risk of Bias Assessment Tool (PROBAST) was applied, which was developed in 2019, including bias risk assessment and applicability assessment (16, 17). It includes four domains: participants, predictors, results, and analysis. Each is rated as “low,” “high,” or “unclear” risk of bias based on two to nine signature questions answered with “yes/probably yes,” “no/probably no,” or “no information.” A domain is rated “high” if any signature question is “no/probably no”; “low” if all are “yes/probably yes”; and “unclear” if at least one is “no information” and the rest are “yes/probably yes.” According to the evaluation results of each field, the bias risk and applicability of the prediction model are obtained. The evaluation of PROBAST is carried out independently by two researchers, and in case of disagreement, a third party was consulted.

#### 2.1.6 Data synthesis and statistical analysis

A meta-analysis of the area under the curve (AUC) values from the validated models was conducted using Stata 15.0, with specific values and 95% confidence intervals (CI) provided. An AUC range of 0.7 to 0.9 indicates moderate predictive accuracy, while an AUC > 0.9 suggests high diagnostic accuracy. Heterogeneity was tested using the *I*^2^ index and Cochrane Q test. If *p* > 0.05 and *I*^2^ ≤ 50%, the heterogeneity is considered acceptable, and a fixed-effect model is used; otherwise, a random-effects model is employed to combine the effect sizes. A *p* < 0.05 is considered statistically significant. Funnel plot and Egger’s test were used to identify publication bias, with *p* > 0.05 indicating a low likelihood of publication bias. If there was a potential bias, the trim-and-fill method was used to reassess.

### 2.2 Results

#### 2.2.1 Study screening

**Figure 1:**
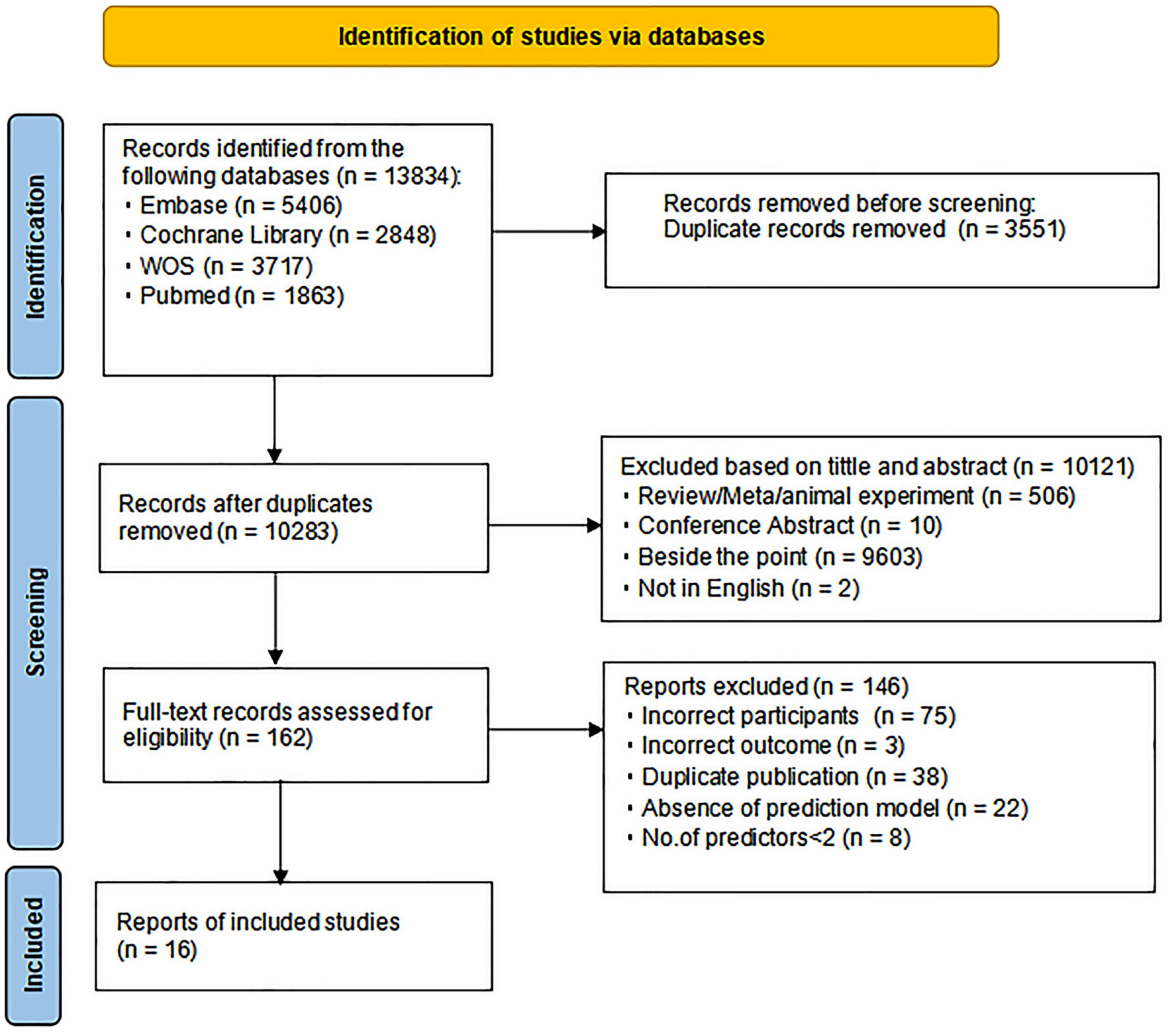
Preferred reporting items for systematic reviews and meta-analyses (PRISMA) flowchart of literature search and selection.

The Preferred Reporting Items for Systematic reviews and Meta-Analyses (PRISMA) 2020 is illustrated in Figure 1, describing the comprehensive search process and results.

A total of 13,834 publications were retrieved from the systematic search in four databases. After the 3,551 duplicate records identified in all databases were removed, the remaining 10,283 were screened by reading titles and abstracts. After the selection, 162 articles were read in full, and finally 16 were included.

#### 2.2.2 Characteristics of the included studies

Table 1 summarizes the design and participant characteristics of the 16 included studies. These studies were published between 2017 and 2024, with nine of them conducted in China. Of the included studies, two were prospective studies, and 14 were retrospective studies. The subjects in four studies were ACS patients, and those in 12 studies were subtypes of ACS. 13 studies were patients with PCI and three were patients with CAG or PCI. Nine studies clearly indicated that the type of interventional procedure was emergency PCI, while the other seven did not make a clear distinction. The sample sizes of these studies varied from 217 to 82,186 individuals. Only five studies specified the hydration protocols and contrast agent protocols, while the remaining studies did not provide detailed information (Supplementary Table S1).

**Table 1:** Overview of basic data of the included studies.

Author year	Country	Study design	Participants	Interventional procedure type	Data source	Main outcome	Total	Cases/Sample size (%)
Stefano (26)	Italy	Prospective cohort study	STEMI	Primary PCI	Policlinico San Matteo in Pavia, Italy, and Centro Cardiologico Monzino in Milan, Italy	CI-AKI	3,736	229/3736 (6.13%)
Yinghua (19)	China	Retrospective study	ACS	PCI	Affiliated Hospital of Xuzhou Medical University and the East Hospital of Xuzhou Medical University	CI-AKI	939	D:69/722
Benjamin (23)	America	Retrospective cohort study	STEMI	Primary PCI	UT Methodist Hospital	AKI	840	/
Yuhei (27)	Japan	Retrospective observational study	STEMI	Primary PCI	Miyazaki Medical Association Hospital	AKI	908	77/908 (8.48%)
Akaphol (24)	Thailand	Retrospective cohort	STEMI	Primary PCI	Central Chest Institute of Thailand,a tertiary care hospital	AKI	1,617	195/1617 (12.06%)
Pei-Chun (30)	China	Retrospective data	ACS	PCI	Taiwan National Health Insurance Research Database	AKI	82,186	3829/82186 (4.66%)
Amir (33)	Iran	Retrospective study	ACS	PCI	Tehran Heart Center	AKIN	4,592	646/4592 (14.07%)
Hang (20)	China	Retrospective analysis	STEMI	Emergency PCI	Xuzhou Medical University Hospital	CI-AKI	542	74/542 (13.65%)
Faysal (28)	Turkey	Retrospective study	STEMI	Primary PCI	Van Training and Research hospital	CIN	2,289	219/2289 (9.57%)
Kai (21)	China	Retrospective study	NSTE-ACS	PCI	The Affiliated Hospital of Xuzhou Medical University	CI-AKI	1,156	168/1156 (14.53%)
Yue (22)	China	Retrospective study	ACS	PCI	Atherosclerotic cardiovascular disease (ASCVD) database-Affiliated Hospital of Xuzhou Medical University	CI-AKI	1,073	213/1073 (19.85%)
Sukrisd (25)	Thailand	Retrospective cohort	STEMI	Primary PCI	Central Chest Institute of Thailand (CCIT)	CIN	217	43/217 (19.82%)
Kai-yang (32)	China	Prospective observational study	STEMI/NSTE-ACS	Emergent PCI	Guangdong general hospital	CIN	692	55/692 (7.95%)
Hui (29)	China	Retrospective analysis	STEMI	Primary CAG/PCI	D: the First Affiliated Hospital of Xinxiang Medical College; V: the First Affiliated Hospital of Henan Polytechnic University	AKI	452	D:57/364
Ling (31)	China	Retrospective cohort study	AMI	CAG/PCI	Changzhou No.2 People’s Hospital of Nanjing Medical University	CI-AKI	1,495	226/1495 (15.12%)
Xuejun (18)	China	Retrospective study	AMI	CAG/PCI	D: central branch of The Affiliated Changzhou No.2 people’s Hospital of Nanjing Medical University; V: Yanghu branch of hospital	CI-AKI	920	164/920 (17.83%)

STEMI, ST-elevation myocardial infarction; pPCI, primary Percutaneous Coronary Interventions; ACS, acute coronary syndromes; CAG, coronary angiography; PCI, percutaneous coronary intervention; AMI, acute myocardial infarction; T2DM, type 2 diabetes mellitus; NSTE-ACS, non-ST-elevation acute coronary syndrome, DM, diabetes mellitus; D, derivation; V, validation; CI-AKI, contrast-induced acute kidney injury; AKI, acute kidney injury; AKIN, acute kidney injury necrosis; CIN, contrast-induced nephropathy; SCr, serum creatinine.

Table 2 provides details about the predictive models used in the included studies. In the realm of model development, 14 studies have employed multivariate logistic regression analysis, whereas two studies have implemented machine learning methodologies. As for the method of variable selection, nine studies used univariate analysis to select the factors related to CI-AKI in ACS patients, and then used multivariate regression analysis to select independent predictive factors. In two of the included studies, a univariate analysis was carried out, LASSO regression was utilized to identify potential predictors, and subsequently, multivariate logistic regression was applied to develop the model. In the other two studies, LASSO regression was utilized to identify predictors, followed by multivariate logistic regression for modeling. Of the 16 included studies, at most 15 predictors were included, and at least three predictors were included. The most frequently utilized predictive factors across the studies were age, serum creatinine (SCr) and left ventricular ejection fraction (LVEF) both appearing in each of the seven models. Additionally, estimated glomerular filtration rate (eGFR) and use of intra-aortic balloon pump (IABP) were commonly used in eight and five models, respectively. Other predictors included diabetes mellitus(DM), multivessel disease, chronic kidney disease(CKD), hypotension, hemoglobin, and highly sensitive C-reactive protein(hsCRP), among others.

**Table 2:** Overview of the information of the included prediction models.

Author year	Variable selection	Model development method	Calibration method	Clinical application	Validation method	Final predictors	Model performance (AUC/C-statistic)	Model presentation
Stefano (26)	Univariate analysis and multivariate logistic regression analysis	Multivariable logistic regression model	Hosmer-Lemeshow test	NI	External validation	Killip class (II, III), Killip class (IV), Diabetes, Anterior STEMI, Age > 75 years, eGFR<60 ml/min/1.73m^2^	D:0.8379(0.802-0.8738); V:0.84	Risk score
Yinghua (19)	Univariate and multivariate regression analyses	Multivariate regression analysis	Hosmer-Lemeshow test, Calibration plot	DCA	Internal(self-sampling method) and external validation	Age, eGFR, TyG index, PNI	D:0.785(0.729-0.841); V:0.802(0.699-0.905)	Nomogram model
Benjamin (23)	Backward selection	Multivariable logistic regression model	Hosmer-Lemeshow test	NI	Internal cross-validation	Age, history of CKD, eGFR, LVEF, LVEDP, whether the patient was hypotensive, whether the patient received an IABP	D:0.77(0.70-0.83); V:0.76(0.70-0.82)	A web-based tool
Yuhei (27)	Stepwise backward	Multivariable logistic regression model	Hosmer-Lemeshow test	NI	Internal validation	Blood sugar(BS) ≥200 mg/dL, high-sensitivity troponin I(hsTnI) >1.6 ng/dL (normal upper limit×50), Albumin ≤3.5 mg/dL, eGFR <45 mL/min/1.73 m^2^	D:0.754(0.733-0.846); V:0.754(0.644-0.839)	Risk score
Akaphol (24)	Backward elimination	Multivariable logistic regression model	Hosmer-Lemeshow test, Calibration plot	NI	Internal validation	Age, baseline creatinine, LVEF < 40%, multi-vessel pPCI, treated with thrombus aspiration, inserted IABP, pre and intra-procedural cardiogenic shock, congestive heart failure	D:0.78(0.75-0.82); V:0.75(0.72-0.79)	An online web
Pei-Chun (30)	Clinically relevant variables	Multivariable logistic model	NI	NI	Internal validation	Age, DM, ventilator use, prior AKI, number of intervened vessels, CKD, IABP use, cardiogenic shock	D:0.874(0.868-0.881); V:0.8624(0.8515-0.8733)	ADVANCIS Score
Amir (33)	Lasso, SHAP	Machine learning(NB, LR, CB, LMP, RF)	NI	NI	Internal validation (five-fold cross-validation)	LVEF, FPG, creatinine, mean creatinine, eGFR	AUC=0.775	Random Forest model
Hang (20)	Univariate analysis, LASSO and multivariate logistic regression analysis	Multivariate logistic regression analysis	Hosmer-Lemeshow test, Calibration curves	DCA	Internal validation (Bootstrap)	DM, LVEF, SII,NT-proBNP, hsCRP	D:0.84(0.790-0.890); V:0.844(0.762-0.926)	Nomogram model
Faysal (28)	Univariate analysis,Lasso and multivariate logistic regression analysis	Multivariable logistic regression analysis	Calibration plot	NI	Internal validation (A bootstrap of 200 replicates)	Age, Hypertension, Hemoglobin, eGFR, Albumin, SIIRI, LVEF, Lesion length, Pain-to-balloon time	AUC=0.97	Nomogram model
Kai (21)	LASSO regression and multivariable logistic regression analysis	Multivariate logistic regression analysis	Hosmer–Lemeshow test, Calibration plot	DCA	Internal validation (Bootstrap internal verification method)	Age >75, LVEF, DM, FAR, hsCRP, lymphocyte count	D:0.835(0.800-0.871); V:0.767(0.711-0.824)	Nomogram model
Yue (22)	LASSO regression and multivariate analyses	Multivariate logistic regression analysis	Calibration curves	DCA	Internal validation (Bootstrap self-sampling method)	Subtypes of ACS, age>75, multivessel coronary artery disease, hyperuricemia, LDL-C, TyG index, eGFR	D:0.811(0.766-0.844); V:0.773(0.712-0.829)	Nomogram model
Sukrisd (25)	Univariate analysis and multivariate logistic regression analysis	Multivariable logistic regression analysis	Hosmer-Lemeshow, Calibration plot	NI	Internal validation(1,000 replicates bootstrapped sampling)	Ejection fraction < 40%, Triple-vessel disease, Use of IABP	D:0.83(0.76-0.90); V:0.77(0.68-0.85)	Risk Stratification Score
Kai-Yang (32)	Univariate analysis and multivariate logistic regression analysis	Multivariate logistic regression analysis	Hosmer-Lemeshow test	NI	Internal validation (bootstrap method)	Age>75, baseline SCr>1.5 mg/dl, hypotension, the use of IABP	V:0.828(0.737-0.920)	Risk score
Hui (29)	Univariate logistic regression analysis and multivariate logistic regression analysis	Multivariate logistic regression analysis	Hosmer-Lemeshow test, Calibration chart	NI	External validation	Age > 72, ejection fraction of no more than 40%, baseline SCr > 102.7 mmol/L, RDW > 13.15, MDCLs	D:0.721(0.652-0.790); V:0.731(0.624-0.838)	Risk score
Ling (31)	Boruta algorithm	Machine learning(DT, SVW, RF, KNN, NB, GBM)	NI	NI	Internal validation Ten-fold cross-validation	Neutrophil percentage, age, Free triiodothyronine, Preoperation hypotension, SCr, Hemoglobin, LDL-C, Total triglycerides, Brain natriuretic peptide, WBC, HDL-C, Heart rate, BMI, Cardiac troponin I, SBP	D:1.000(1.000-1.000); V:0.82(0.76-0.87)	Random Forest model
Xuejun (18)	Univariate logistic regression analysis and multivariate logistic regression analysis	Multivariate logistic regression analysis	Hosmer-Lemeshow test, Calibration plot	NI	Internal and external validation	Hemoglobin, contrast volume >100ml, hypotension before the procedure, eGFR, logBNP, age	D:0.775(0.732-0.819); V:0.715(0.631-0.799)	Nomogram model

NI, no information; D, derivation; V, validation; LVEF, left ventricular ejection fraction; LVEDP, left ventricular end diastolic pressure; IABP, intra-aortic balloon pump; DCA, decision curve analysis; eGFR, estimated glomerular filtration rate; DM, diabetes mellitus; SII, immune-inflammatory index; NT-proBNP, N-terminal pro-brain natriuretic peptide; hsCRP, highly sensitive C-reactive protein; SIIRI, Systemic immune-inflflammation response index; FAR, fibrinogen-to-albumin ratio; LDL-C, low-density lipoprotein cholesterol; TyG, triglyceride-glucose; SCr, serum creatinine; RDW, red blood cell distribution width; MDCLs, middistal segment culprit lesions; HDL-C, High-density lipoprotein cholesterol; BMI, Body mass index; PNI, prognostic nutritional index; SBP, systolic blood pressure; WBC, white blood cell; BNP, B-type natriuretic peptide.

Among the included studies, 12 studies underwent internal validation, while two studies underwent external validation. Yinghua et al.’s model and Xuejun et al.’s model contained both internal and external validation, which shows that their research stands out (18, 19).

Discrimination, as assessed by the C-statistic, is the most critical metric for evaluating the predictive performance of a model. Reported C statistical values ranged from 0.715 to 1.000, which shows good prediction performance (AUC>0.700). Calibration was reported in 13 models, with the Hosmer-Lemeshow test being the most commonly used method. In addition, a total of three studies did not report calibration information. In terms of clinical application, only four out of the 16 studies addressed this aspect, while the remaining studies did not mention it (19–22). In the included studies, the models presented different forms, mainly risk scores and nomograms. Additionally, two models used website, the other two were Random Forest (RF) models.

#### 2.2.3 Risk of bias and applicability assessment

We used the PROBAST to assess the risk of bias and applicability of all 16 included researches (Table 3). All studies were assessed as having a high risk of bias, indicating methodological problems during development or validation.

**Table 3:** PROBAST results of the included studies.

Author year	Study type	ROB				Applicability			Overall	
		Participants	Predictors	Outcome	Analysis	Participants	Predictors	Outcome	ROB	Applicability
Stefano (26)	B	−	+	+	−	+	+	+	−	+
Yinghua (19)	B	−	+	+	−	−	+	+	−	−
Benjamin (23)	A	−	+	+	−	+	+	+	−	+
Yuhei (27)	A	−	+	+	−	+	+	+	−	+
Akaphol (24)	A	−	+	−	−	+	+	+	−	+
Pei-Chun (30)	A	−	+	+	−	+	+	+	−	+
Amir (33)	A	−	+	−	−	+	+	+	−	+
Hang (20)	A	−	+	+	−	−	+	+	−	−
Faysal (28)	A	−	+	+	−	+	+	+	−	+
Kai (21)	A	−	+	+	?	+	+	+	−	+
Yue (22)	A	−	+	+	?	−	+	+	−	−
Sukrisdi (25)	A	−	+	+	−	+	+	+	−	+
Kai-yang (32)	A	−	+	−	−	+	+	+	−	+
Hui (29)	B	−	+	−	−	+	+	+	−	+
Ling (31)	A	−	+	−	−	+	+	+	−	+
Xuejun (18)	B	−	+	+	−	+	+	+	−	+

PROBAST, Prediction model Risk of Bias Assessment Tool; ROB, Risk of Bias; A indicates “development only”; B indicates “development and validation in the same publication”; + indicates low ROB/low concern regarding applicability; - indicates high ROB/high concern regarding application? Indicates unclear ROB/unclear concern regarding applicability.

In the participant domain, all studies were identified as having a high risk of bias, mainly due to the type of study and the risk of disease in the subjects studied. In the predictor domain, all of the studies were determined to have a low risk of bias. Among the outcome domains of the 16 studies, five had a high risk of bias due to the inclusion of predictors in the outcome definition, while the remainder exhibited a low risk of bias. In the analysis domain, the risk of bias assessment results of two studies in this field were “unclear,” and the rest were “high risk of bias.” The sample size is reasonable according to whether EPV (events per variable) is greater than or equal to 20. In three articles, it was not possible to judge whether the sample size was reasonable because EPVs from model development could not be calculated or validation set data were not reported (23–25). Six studies partially or fully converted continuous variables to categorical variables (24–29), nine studies did not have enough information to determine whether the methods for handling missing data were appropriate (18, 20–22, 25, 27–30), three studies handled the missing data inappropriately (23, 24, 31). In the screening of predictors, 11 of the 16 studies were based on univariate analysis (18–20, 23–29, 32). Almost all of the studies provided no information about the complexity of the data. Two studies did not perform calibration (30, 33). The internal validation methods of four studies included only random split validation or no internal validation (26, 27, 29, 30). Three studies did not provide information on the coefficients of the predictors in the multivariate regression model (19, 31, 33).

The assessment of the applicability of the 16 included studies included three aspects: participants, predictors, and outcomes. In the participant domain, three studies were considered to have a high risk of applicability owing to the participants being limited to specific populations with ACS. Both the predictor and outcome domains were of low risk of applicability.

#### 2.2.4 Meta-analysis of derivation models

**Figure 2:**
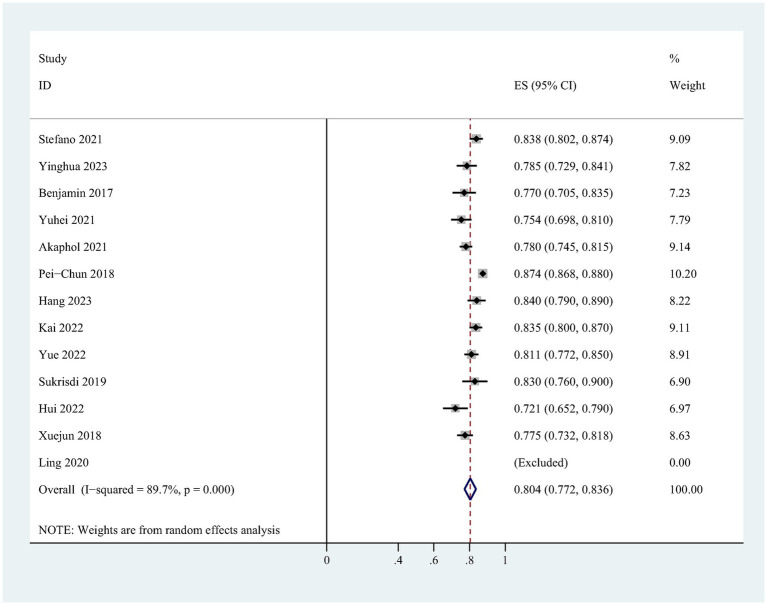
Forest plot of the random effects meta-analysis of pooled AUC estimates for derivation models.

There were discrepancies in the details of the included models and the information provided was incomplete. Only 13 studies met the synthetic criteria. Heterogeneity test results showed that I^2^ = 89.7% (*p* = 0.000), indicating a high degree of heterogeneity between studies, so the random effects model was used for meta-analysis. The pooled AUC estimate of the developed models was 0.804 (95% CI: 0.772–0.836; Figure 2). Sensitivity analyses confirmed the robustness of the results (Supplementary Figure S1). Egger’s test (*t* = −6.90, *p* = 0.000) showed a significant publication bias, which was also confirmed by funnel plots (Supplementary Figure S2), but the trimming and filling method did not fill in the studies, indicating that the meta-analysis results were robust.

#### 2.2.5 Meta-analysis of validation models

**Figure 3:**
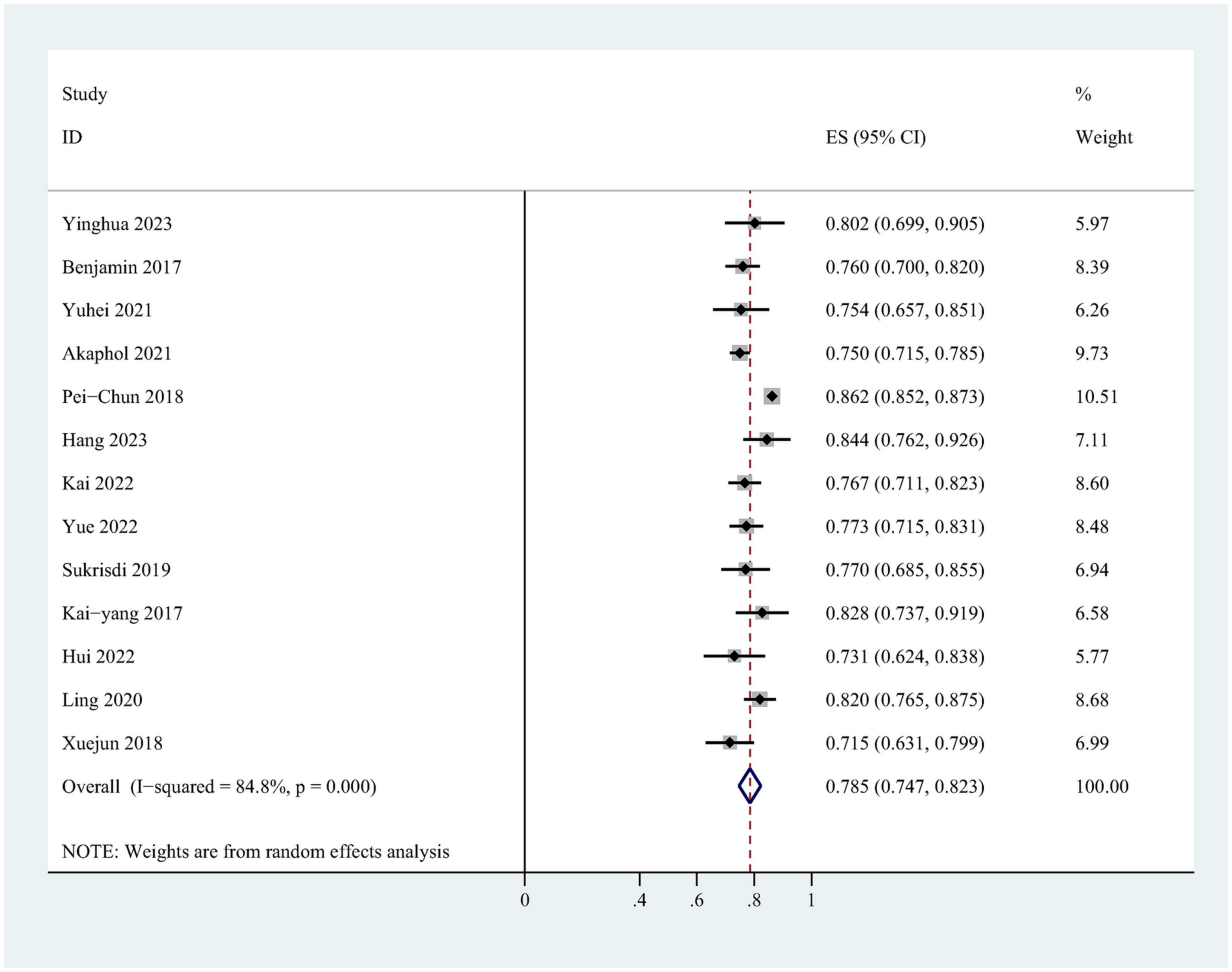
Forest plot of the random effects meta-analysis of pooled AUC estimates for validation models.

The validation model utilized a random-effects model to compute the combined AUC, resulting in 0.785 (95% CI: 0.747–0.823; Figure 3). The *I*^2^ = 84.8% (*p* = 0.000), suggesting a high degree of heterogeneity among the researches. Sensitivity analysis showed that all the values were within the estimated 95% CI, indicating the robustness of the results (Supplementary Figure S3). Egger’s test (*t* = −4.11, *p* = 0.002) showed a significant publication bias, which was also confirmed by funnel plots (Supplementary Figure S4). However, the trimming and filling method did not work, indicating that the results were robust.

### 2.3 Discussion

This study systematically reviewed the risk prediction models for CI-AKI in patients with ACS after PCI or CAG, aiming to screen for prediction models suitable for clinical use. 16 risk prediction models were included in this study, and the modeling method was mainly logistic regression analysis, and only one quarter of the models were externally validated. The AUC values reported range from 0.715 to 1.000, indicating that these models have good predictive performance. However, upon assessment with PROBAST, all included studies were rated as having a high risk of bias, which would weaken their practical utility in clinical applications. In the development models, the pooled AUC was 0.804 (95% CI: 0.772–0.836), and 0.785 (95% CI: 0.747–0.823) in the validation model.

#### 2.3.1 Prediction factor analysis

In this study, age, eGFR, SCr, LVEF, and the use of IABP were commonly used predictors. The results are consistent with Mehran’s models (34). Advanced age was correlated with a greater incidence of CIN, aligning with the findings of a meta-analysis (35). The reason is that the kidney metabolism of elderly patients is slow, and after the application of a contrast agent, it is more likely to cause renal hemodynamic changes, renal medulla hypoxia, renal tubular poisoning and other conditions (36, 37). Therefore, clinical attention should be paid to the elderly, alert to the occurrence of postoperative kidney injury.

The eGFR is an evaluation index of renal function, representing the basic renal function and compensatory ability. The lower the value, the higher the risk of occurrence and progression of renal damage. SCr is the most commonly used marker for assessing renal function; however, it is prone to variations due to factors such as age, sex, race, and protein consumption, and it has limited specificity. In contrast, cystatin C (CyC) offers greater sensitivity and can swiftly identify early (within 24 h) acute renal function changes with high sensitivity and dependability. The elevation in CyC levels typically peaks within 24 h following exposure to a nephrotoxic agent (38). It can not only early diagnose CI-AKI at 24 h after CM exposure, but also predict the occurrence of future major adverse events (MAE). Therefore, CyC levels can be measured at an early stage for early intervention.

Patients exhibiting a low left ventricular ejection fraction (LVEF) are at an increased risk for developing CI-AKI. This heightened risk could be attributed to their diminished cardiac output, which may adversely affect tissue and organ perfusion, leading to compromised renal function and a less favorable prognosis (21).

The use of IABP is an indicator of hemodynamic instability and is an independent predictor of CA-AKI (39). Patients with an IABP typically exhibit severe illness and compromised cardiac function. These individuals often experience reduced kidney perfusion and are at an increased risk of kidney injury, a finding that aligns with the observations of Reza et al. (40). The amount of intraoperative contrast agent is also stressed. In 2023, Aiste’s team proposed a simple strategy, building on previous theory, to reduce the amount of contrast used during PCI and thereby reduce the risk for CI-AKI (41). For patients with diabetes, on the one hand, the use of hypoglycemic drugs is essential. In a systematic review, SGLT2-I was found to reduce the risk of developing CI-AKI by 63% in patients with diabetes after CAG or PCI (42). Another study of 646 AMI patients with diabetes found that the use of SGLT2-I reduced the risk of both in-hospital and long-term adverse cardiovascular outcomes (43). On the other hand, monitoring of fasting plasma glucose and glycosylated hemoglobin A1c (HbA1c) is essential. Yu’s group investigated the relationship between Stress hyperglycemia ratio (SHR) and CI-AKI in a ratio of the above two. It was found that both the lowest and highest levels of fasting SHR were significantly associated with an increased occurrence of CI-AKI (44). However, current models rely heavily on static indicators (age, eGFR, etc.) Future efforts should focus on integrating mechanistic biomarkers that reflect the dynamic disease trajectories post-AMI (45) and personalized risk, such as novel RNA biomarkers (HCG15 and Morrbid) (46) or even gut microbiota (47), to achieve more precise prediction.

The included models contain preoperative and intraoperative predictors. Risk assessment using preoperative predictors helps identify high-risk CI-AKI patients early, facilitating preoperative planning and guiding the application of intraoperative preventive measures. For intraoperative predictors, all relevant data are available after surgery, allowing for accurate risk prediction. This final risk assessment is superior to a single preoperative assessment and can directly guide enhanced monitoring and precise management of high-risk patients. Therefore, preoperative and intraoperative predictors should be integrated to establish a dynamic assessment system, enabling full-course risk management from preoperative early warning to postoperative precise intervention.

#### 2.3.2 Deficiencies and suggestions of the models

There are some shortcomings in the existing prediction models.

In the data source, only two of the included studies were prospective, while the rest were retrospective. Compared with prospective studies, retrospective studies may overstate model performance: they may involve data collection with known outcomes, easily introducing information unavailable in real-world model applications; data mostly come from single-center medical institutions, which may introduce selection bias due to specific selection criteria of the institutions and diagnostic-therapeutic protocols. The patients in these institutions have more severe and typical conditions, while mild cases or negative outcomes may be excluded. Improper handling of missing values in the data can easily introduce bias. Consequently, model performance may only be suited to historical data, and its generalizability is questionable. Most of the included studies were conducted in Asia. Since Asian populations differ from other regions in disease epidemiology and treatment strategies, the international application and promotion of the studies may be limited. Therefore, future studies should conduct multi-center, cross-regional prospective cohort studies with more geographically representative populations to verify its global generalizability. There is no unified standard for CI-AKI currently (48–55), and the exclusion criteria only excluded patients with severe renal insufficiency or requiring dialysis, but not those with chronic renal insufficiency, which may overestimate the incidence of CI-AKI and bring bias. Researchers should unify all key research indicators based on the standard definitions in the guidelines. This will ensure the research process is standardized and the results are comparable. Nearly half of the 16 included studies failed to clearly specify the type of PCI procedure involved. Since patients undergoing emergency PCI have a higher risk of CI-AKI than those undergoing elective PCI (56, 57). Even if the models have good performance, the ambiguity in procedure type may limit the applicable scenarios of these models. Future studies should clearly distinguish between emergency and elective procedures for more accurate evaluation of model applicability. Although five studies have indicated that both their hydration protocols and contrast agent protocols were consistent with the Kidney Disease Improving Global Outcomes (KDIGO) recommendations (58), other studies should also specify the protocols inorder to facilitate the exploration of heterogeneity.

In the outcome domain, five studies incorporated predictors into the outcome definition. This could lead to models utilizing this information during training and prediction, resulting in overestimation of model performance, model overfitting, and an inability to accurately assess the importance of variables.

In the statistical analysis, some studies converted continuous variables into categorical variables, which led to the loss of data information. This may fail to fully reflect the true characteristics of variables and reduce the accuracy of predictions. Meanwhile, among the 16 included studies, nine studies did not report information on missing data and data complexity. This reduces the transparency and reproducibility of the research, making it difficult to determine the potential impact of missing data on the results and hindering other researchers from verifying or drawing on the research. These issues have an adverse impact on the scientific evaluation and practical application of prediction models. Most of the studies used univariate analysis to screen predictors. Univariate analysis may ignore the interaction and confounding effects between variables, thereby introducing bias and increasing the risk of overfitting (59).

Currently, machine learning has gradually become a hot spot, which can effectively improve the predictive capabilities of models to deal with complex issues by using a variety of algorithms. In this review, Amir et al. and Ling et al. used machine learning to build a variety of prediction models, but in the end, the random forest model proved to have the best performance (31, 33). Moreover, although all studies showed good prediction performance, only four studies conducted external validation, which limits their clinical generalization. For instance, the model built by Ling et al. using machine learning methods showed perfect performance (AUC = 1.000), but a lack of external verification, in addition, the calibration method was not explained, which made the overfitting problem of the model not reasonably solved (31). In terms of clinical application, only four studies evaluated it using decision curve analysis (DCA). However, these studies did not clearly illustrate whether the models truly improved clinical conditions and prognosis, such as mortality, dialysis requirement, or length of hospital stay. On one hand, most prediction models stayed at the stages of development and internal validation. Few explored their impact on clinical outcomes. On the other hand, prediction tools including nomograms or web-based calculators had not been tested in real-world settings and lacked cost-effectiveness evaluations. Therefore, future study should shift focus from model development to implementing existing high-quality models, verifying their clinical utility, and conducting sound cost-effectiveness assessments.

Overall, to enhance the performance and popularity of the prediction models, future studies should develop models based on multi-center, cross-regional prospective cohort studies with more geographically representative populations. To improve the clinical utility, future studies should shift focus from model development to implementing existing high-quality models. Additionally, external validation is required to enhance the model’s generalization ability, with the core being to assess the model’s stability and applicability in new data. An independent dataset should be selected that differs from the source of the development cohort (e.g., different centers, different time periods, different population characteristics) but is consistent with the target population, and it must include all input variables and outcome indicators of the model. This ensures that external validation can truly reflect the model’s generalization ability and application reliability in diverse real-world scenarios. At the same time, when screening variables, it is recommended to avoid only basing on univariate analysis, but to match traditional regression algorithms with machine learning algorithms and professional knowledge background. Furthermore, future study should shift focus from model development to implementing existing high-quality models, verifying their clinical utility, and conducting cost-effectiveness assessments.

#### 2.3.3 Prevention is more important than treatment

There is no specific treatment method in clinical practice, and prevention is far more important than treatment. The KDIGO recommends that for patients at risk of CI-AKI, volume expansion with either isotonic sodium chloride solution or sodium bicarbonate solution should be performed, rather than no volume expansion (58, 60). Hydration therapy is to expand the volume of the patient, dilute the concentration of contrast agent in the kidney, reduce the contact between the contrast agent and the kidney and reduce the viscosity of urine, thereby accelerating the excretion of contrast agent. It is currently the most internationally recognized preventive measure. However, the method of hydration varies according to the individual condition of the patient. Intravenous fluid should be administered at a rate of ≥1.0–1.5 mL/kg/h for 3–12 h before and 6–12 h after contrast media exposure to maintain a urine output of at least 150 mL/h, thereby reducing the risk of CI-AKI (58, 61). Contrast nephropathy (CIN) or CI-AKI is an iatrogenic acute kidney injury observed after intravascular injection of contrast material (CM) for diagnostic procedures or therapeutic angiographic interventions (10). According to guideline recommendations, iso-osmolar or low-osmolar iodinated contrast media should be used instead of high-osmolar ones (58). Choosing the appropriate contrast agent and minimizing the amount used, while still ensuring accurate diagnosis and effective treatment, are prudent strategies to prevent CI-AKI.

#### 2.3.4 Limitations

The systematic review has several potential limitations. Firstly, the present study had a high degree of heterogeneity. Secondly, there was significant publication bias in this meta-analysis. Consequently, the pooled AUC may not be adequately representative. Thirdly, most of the included studies were conducted in Asia, which limits the international generalizability of the findings. Moreover, only English-language publications were included, meaning relevant studies might have been omitted due to language barriers. Future studies can collaborate with teams with multilingual backgrounds and expand the language scope of retrieval to more comprehensively integrate various research results and reduce the impact of bias.

### 2.4 Conclusion

A total of 16 studies on risk prediction models for CI-AKI after PCI or CAG in patients with ACS were included in this review. The developed models exhibited a pooled AUC of 0.804 (95% CI: 0.772–0.836), while the validation models demonstrated a pooled AUC of 0.785 (95% CI: 0.747–0.823). However, all studies had a high risk of bias and two studies raised concerns about applicability. A quarter of the studies lacked external validation, and the clinical application of the model needs further validation. Thus, no existing model of CI-AKI after CAG or PCI with ACS can currently be recommended for routine use. Based on current data, the clinical utility of these models should be postponed until the development of methodologically robust, externally validated tools tested in prospective multicenter settings, with standardized definitions of CI-AKI and clinical impact assessments. Furthermore, researchers should follow PROBAST to improve the quality of prediction models and provide greater clinical value.

## Data Availability

The original contributions presented in the study are included in the article/Supplementary material, further inquiries can be directed to the corresponding author/s.
