# Supplementary figures and images for: Risk prediction models for contrast-induced acute kidney injury in patients with acute coronary syndromes: a systematic review and meta-analysis
Source: Front Med (Lausanne). 2025 Sep 18;12:1629369. doi: 10.3389/fmed.2025.1629369 (PMC12488721; doi:10.3389/fmed.2025.1629369)

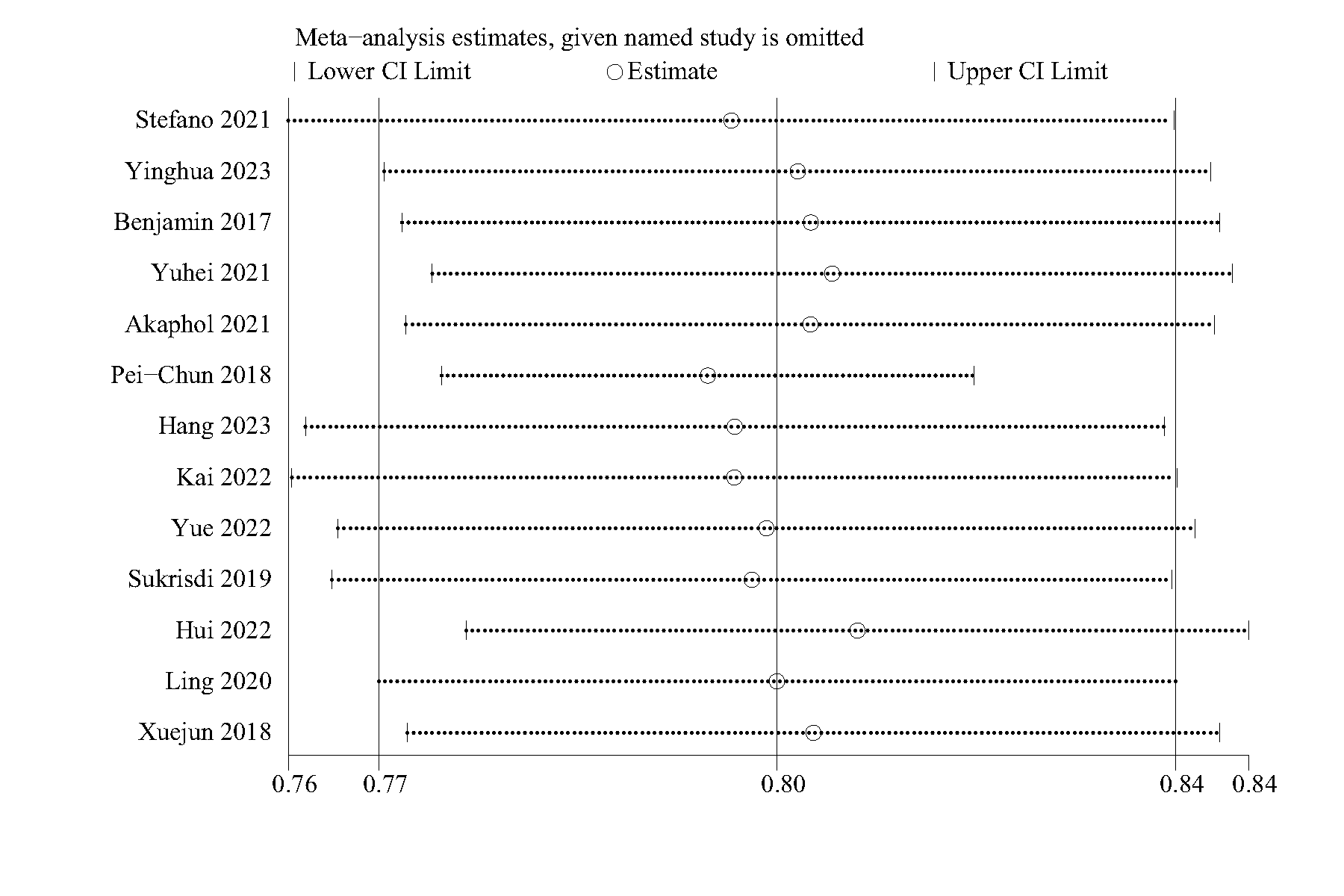

Supplement: Supplementary file 1 [file Image_1.TIF]

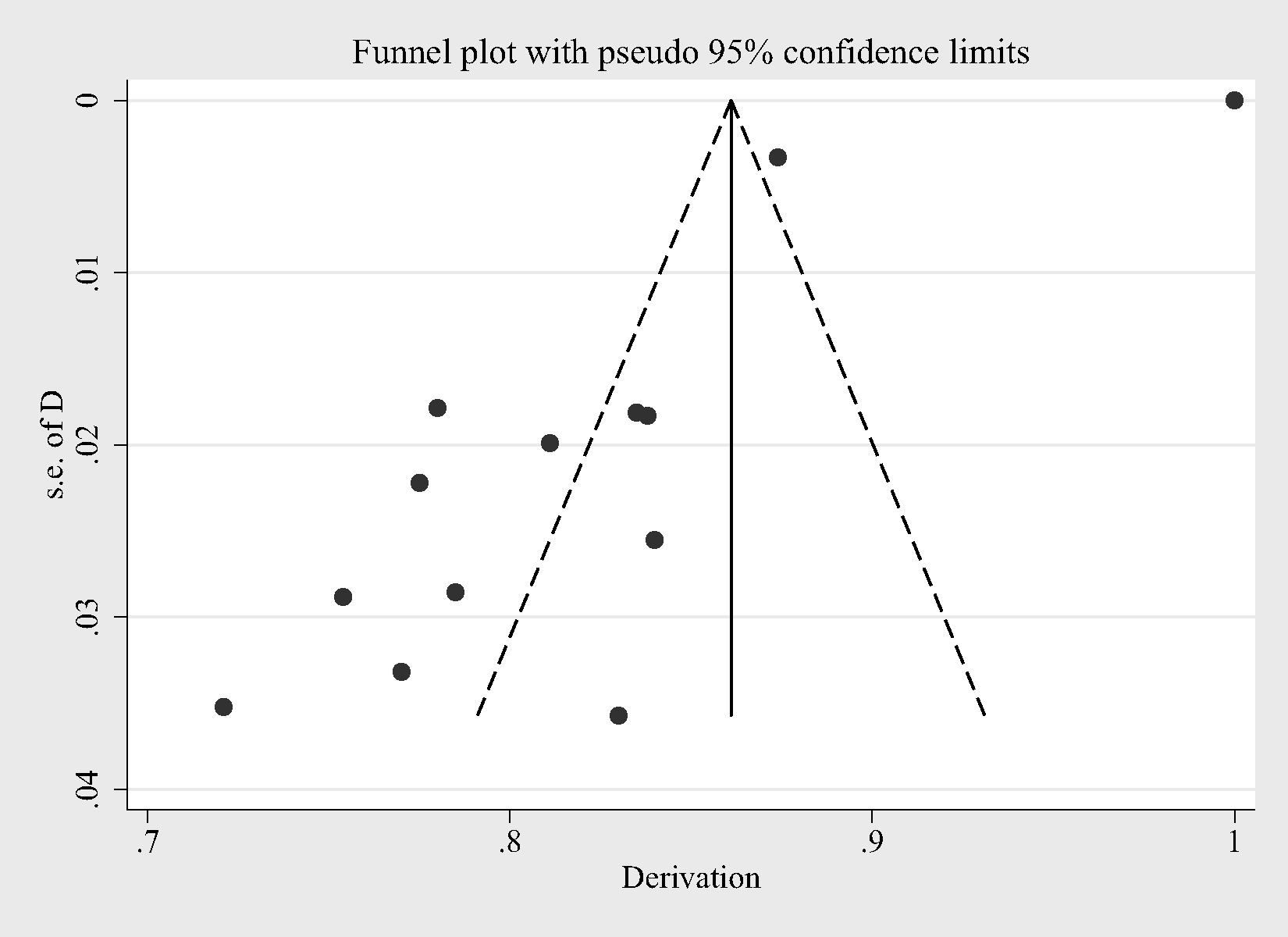

Supplement: Supplementary file 2 [file Image_2.TIF]

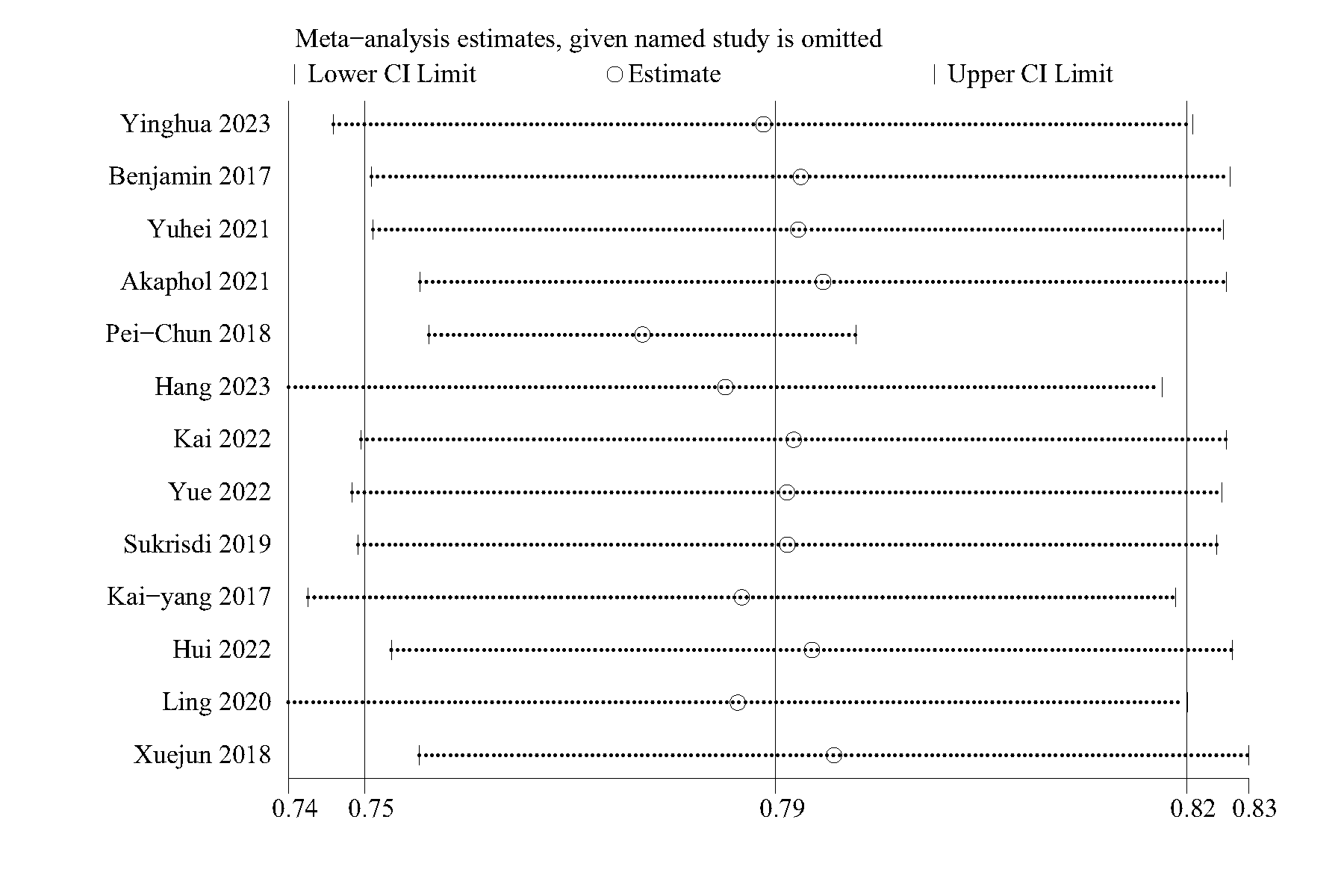

Supplement: Supplementary file 3 [file Image_3.TIF]

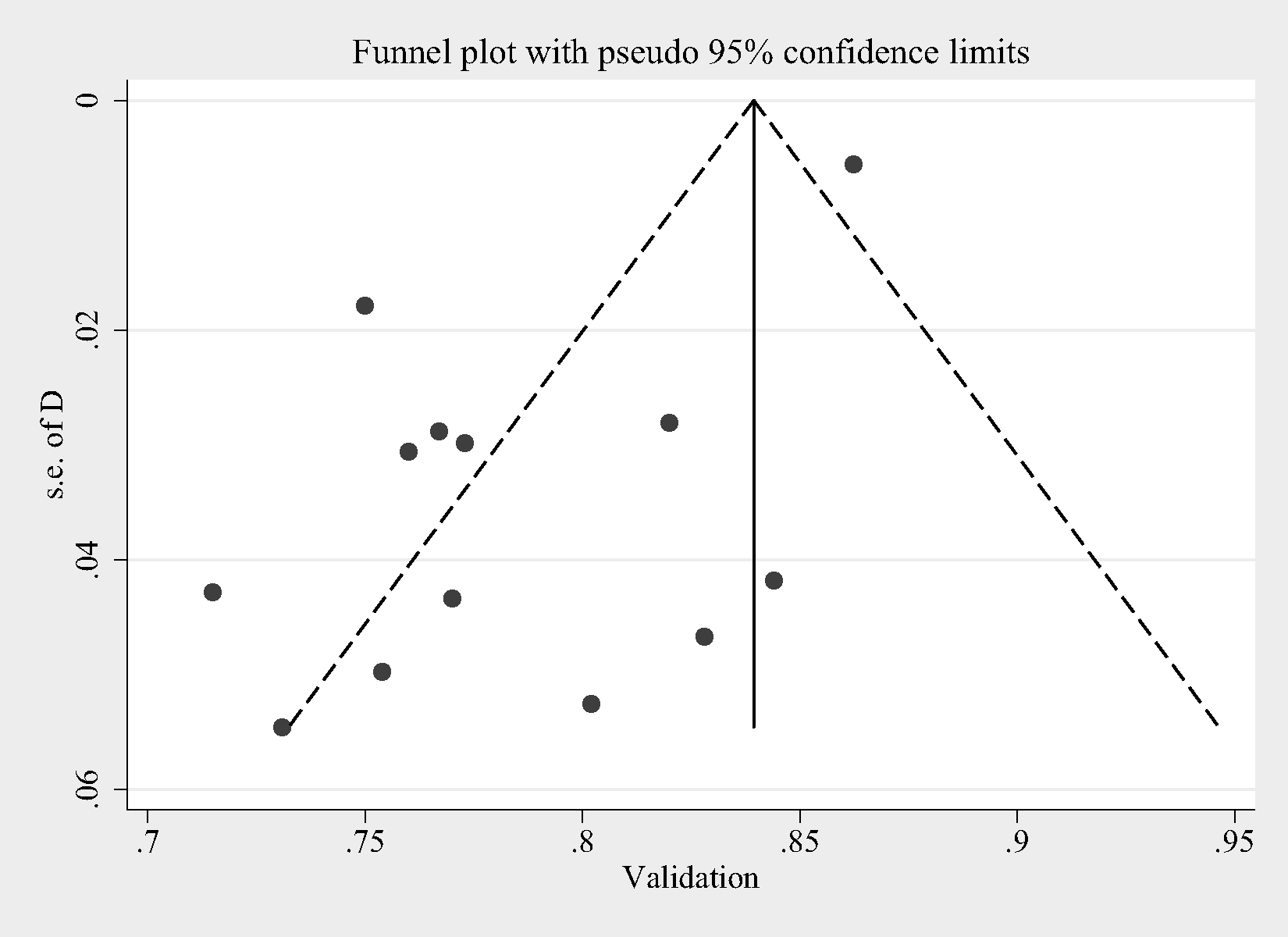

Supplement: Supplementary file 4 [file Image_4.TIF]
