# Supplementary material for: Risk prediction models for contrast-induced acute kidney injury in patients with acute coronary syndromes: a systematic review and meta-analysis
Source: Front Med (Lausanne). 2025 Sep 18;12:1629369. doi: 10.3389/fmed.2025.1629369 (PMC12488721; doi:10.3389/fmed.2025.1629369)
Supplement: Supplementary file 5 [file Table_1.DOCX]

Supplementary Material

# Supplementary Data

**Search Strategy**

2024/5/18

Embase 5406

('acute coronary syndrome'/exp OR 'acute coronary syndrome' OR 'unstable angina pectoris'/exp OR 'unstable angina pectoris' OR 'heart infarction'/exp OR 'heart infarction' OR 'acute heart infarction'/exp OR 'acute heart infarction' OR 'st segment elevation myocardial infarction'/exp OR 'st segment elevation myocardial infarction' OR 'non st segment elevation myocardial infarction'/exp OR 'non st segment elevation myocardial infarction' OR 'acute coronary syndrome':ab,ti OR 'unstable angina pectoris':ab,ti OR 'angina, unstable':ab,ti OR 'myocardial infarction':ab,ti OR 'acs':ab,ti OR 'ua':ab,ti OR 'uap':ab,ti OR 'unstable angina':ab,ti OR 'acute heart infarction':ab,ti OR 'ami':ab,ti OR 'acute myocardial infarction':ab,ti OR 'stemi':ab,ti OR 'st elevation myocardial infarction':ab,ti OR 'st elevated myocardial infarction':ab,ti OR 'st-segment elevation myocardial infarction':ab,ti OR 'ste-acs':ab,ti OR 'st-segment elevation acute coronary syndrome':ab,ti OR 'nstemi':ab,ti OR 'non stemi':ab,ti OR 'non-st elevated myocardial infarction':ab,ti OR 'non-st-segment elevation myocardial infarction':ab,ti OR 'nste-acs':ab,ti OR 'non-st-segment elevation acute coronary syndrome':ab,ti OR 'acute coronary syndrome*':ab,ti OR 'acute cardiac infarction':ab,ti OR 'cardia* infarct*':ab,ti OR 'heart infarct*':ab,ti OR 'myocardium infarct*':ab,ti OR 'myocardial infarct*':ab,ti OR 'st elevated mi':ab,ti OR 'non st elevated mi':ab,ti OR 'st elevation mi':ab,ti OR 'non st elevation mi':ab,ti OR 'st segment elevat* myocardial infarction':ab,ti OR 'non st segment elevat* myocardial infarction':ab,ti OR 'st segment elevation heart infarction':ab,ti OR 'non st segment elevation heart infarction':ab,ti OR 'st segment elevation mi':ab,ti OR 'non st segment elevation mi':ab,ti OR 'angina at rest':ab,ti OR 'cardiovascular stroke*':ab,ti OR 'heart attack*':ab,ti) AND ('kidney injury'/exp OR 'kidney injury' OR 'acute kidney failure'/exp OR 'acute kidney failure' OR 'contrast induced nephropathy'/exp OR 'contrast induced nephropathy' OR 'kidney injury':ab,ti OR 'renal injury':ab,ti OR 'acute kidney failure':ab,ti OR 'acute renal failure':ab,ti OR 'renal damage':ab,ti OR 'kidney damage':ab,ti OR 'acute kidney injury':ab,ti OR 'acute renal injury':ab,ti OR 'aki':ab,ti OR 'can':ab,ti OR 'radiographic contrast nephropathy':ab,ti OR 'cin':ab,ti OR 'contrast associated nephropathy':ab,ti OR 'contrast media-induced nephropathy':ab,ti OR 'contrast-media induced nephropathy':ab,ti OR 'renal insufficienc*':ab,ti OR 'kidney insufficienc*':ab,ti OR 'renal failure*':ab,ti OR 'kidney failure*':ab,ti OR 'kidney lesion':ab,ti OR 'renal lesion':ab,ti OR 'contrast agent induced nephropathy':ab,ti OR 'contrast agent induced nephrotoxicity':ab,ti OR 'contrast induced acute renal failure':ab,ti OR 'contrast induced nephrotoxicity':ab,ti OR 'contrast induced nephropathy':ab,ti OR 'contrast induced renal dysfunction':ab,ti OR 'contrast induced renal failure':ab,ti OR 'contrast media induced nephropathy':ab,ti OR 'contrast media induced nephrotoxicity':ab,ti OR 'contrast media induced renal failure':ab,ti OR 'contrast medium induced nephropathy':ab,ti OR 'contrast medium induced nephrotoxicity':ab,ti OR 'contrast medium induced renal failure':ab,ti OR 'contrast nephropathy':ab,ti OR 'contrast nephrotoxicity':ab,ti OR 'contrasting agent-induced nephropathy':ab,ti OR 'radio-contrast nephropathy':ab,ti OR 'radio-contrast-induced nephropathy':ab,ti OR 'radiocontrast nephropathy':ab,ti OR 'radiocontrast-induced nephropathy':ab,ti OR 'rc-induced nephropathy':ab,ti) AND ('risk assessment'/exp OR 'risk assessment' OR 'risk factor'/exp OR 'risk factor' OR 'regression analysis'/exp OR 'regression analysis' OR 'decision tree'/exp OR 'decision tree' OR 'random forest'/exp OR 'random forest' OR 'artificial neural network'/exp OR 'artificial neural network' OR 'machine learning'/exp OR 'machine learning' OR 'neural networks, computer':ab,ti OR 'risk assessment*':ab,ti OR 'risks and benefits':ab,ti OR 'benefits and risks':ab,ti OR 'risk analy*':ab,ti OR 'benefit-risk':ab,ti OR 'benefit risk':ab,ti OR 'risk benefit':ab,ti OR 'risk-benefit':ab,ti OR 'forecast* model*':ab,ti OR 'risk prediction score':ab,ti OR 'risk adjustment':ab,ti OR 'risk evaluation':ab,ti OR 'risk factor*':ab,ti OR 'population* at risk':ab,ti OR 'risk score*':ab,ti OR 'risk prediction model*':ab,ti OR 'risk assessment model*':ab,ti OR 'protection':ab,ti OR 'regression analy*':ab,ti OR 'sregression model analysis':ab,ti OR 'regression model*':ab,ti OR 'regression diagnostics':ab,ti OR 'statistical regression*':ab,ti OR 'relative risk':ab,ti OR 'predictive model*':ab,ti OR 'predictive simulation':ab,ti OR 'prediction model*':ab,ti OR 'prognostic model*':ab,ti OR 'predict* factor*':ab,ti OR 'prognostic factor*':ab,ti OR 'predict* variable*':ab,ti OR 'protect* factor*':ab,ti OR 'decision tree*':ab,ti OR 'random forest*':ab,ti OR 'random decision forest':ab,ti OR 'ann':ab,ti OR 'ann analysis':ab,ti OR 'ann approach':ab,ti OR 'ann method*':ab,ti OR 'ann model*':ab,ti OR 'ann output':ab,ti OR 'ann technique*':ab,ti OR 'ann training':ab,ti OR 'anns':ab,ti OR 'algorithmic neural network':ab,ti OR 'artificial nn':ab,ti OR 'artificial nns':ab,ti OR 'artificial neural network*':ab,ti OR 'computational neural network':ab,ti OR 'support vector*':ab,ti OR 'vector machine*':ab,ti OR 'vector network*':ab,ti OR 'svc':ab,ti OR 'svm':ab,ti OR 'support vector machine':ab,ti OR 'svr':ab,ti OR 'support vector classifi*':ab,ti OR 'support vector regression':ab,ti OR 'machine learning':ab,ti OR 'transfer learning':ab,ti OR 'learning machine*':ab,ti OR 'nomogram':ab,ti OR 'safety assessment':ab,ti OR 'health risk assessment*':ab,ti OR 'benefit-risk assessment*':ab,ti OR 'benefit risk assessment':ab,ti OR 'risk-benefit assessment*':ab,ti OR 'risk benefit assessment':ab,ti OR 'risk factor score*':ab,ti OR 'random forest classification*':ab,ti OR 'connectionist network':ab,ti OR 'connectionist system':ab,ti OR 'neural network*':ab,ti OR 'random forest algorithm*':ab,ti OR 'computer neural':ab,ti OR 'network model*':ab,ti OR 'computational neural':ab,ti OR 'perceptron*':ab,ti OR 'connectionist model*':ab,ti OR 'partin table*':ab,ti OR 'partin nomogram*':ab,ti) AND ('percutaneous coronary intervention'/exp OR 'percutaneous coronary intervention' OR 'transluminal coronary angioplasty'/exp OR 'transluminal coronary angioplasty' OR 'percutaneous coronary intervention*':ab,ti OR 'percutaneous coronary revascularization*':ab,ti OR 'pci':ab,ti OR 'percutaneous transluminal coronary intervention':ab,ti OR 'percutaneous endoluminal stenting':ab,ti OR 'ptas':ab,ti OR 'angioplasty':ab,ti OR 'ptca':ab,ti OR 'angioplasty, balloon, coronary':ab,ti OR 'coronary intervention*':ab,ti OR 'percutaneous coronary':ab,ti OR 'coronary revascularization*':ab,ti OR 'transluminal coronary balloon dilation':ab,ti OR 'balloon dilation':ab,ti OR 'coronary artery':ab,ti OR 'angioplast*':ab,ti OR 'coronary balloon':ab,ti OR 'balloon angioplast*':ab,ti OR 'transluminal balloon':ab,ti OR 'percutaneous transluminal coronary angioplasty':ab,ti OR 'coronary angioplasty':ab,ti OR 'p.t.c.a.':ab,ti OR 'percutaneous coronary transluminal angioplasty':ab,ti OR 'transluminal coronary angioplasty':ab,ti) AND ([article]/lim OR [article in press]/lim) AND [english]/lim AND [humans]/lim

Pubmed 1863

#25 Search: ((((((("Acute Coronary Syndrome"[Mesh]) OR ("Angina, Unstable"[Mesh])) OR ("Myocardial Infarction"[Mesh])) OR ("ST Elevation Myocardial Infarction"[Mesh])) OR (((((((((((((((((((((((((((((((((((((((("unstable angina pectoris"[Title/Abstract]) OR ("Angina, Unstable"[Title/Abstract])) OR ("ACS"[Title/Abstract])) OR ("UA"[Title/Abstract])) OR ("UAP"[Title/Abstract])) OR ("unstable angina"[Title/Abstract])) OR ("Acute heart infarction"[Title/Abstract])) OR ("AMI "[Title/Abstract])) OR ("acute myocardial infarction"[Title/Abstract])) OR ("STEMI"[Title/Abstract])) OR ("ST Elevation Myocardial Infarction"[Title/Abstract])) OR ("ST Elevated Myocardial Infarction"[Title/Abstract])) OR ("ST-Segment Elevation Myocardial Infarction"[Title/Abstract])) OR ("STE-ACS"[Title/Abstract])) OR ("ST-Segment Elevation Acute Coronary Syndrome"[Title/Abstract])) OR ("NSTEMI"[Title/Abstract])) OR ("non STEMI"[Title/Abstract])) OR ("Non-ST Elevated Myocardial Infarction"[Title/Abstract])) OR ("Non-ST-Segment Elevation Myocardial Infarction"[Title/Abstract])) OR ("NSTE-ACS"[Title/Abstract])) OR ("Non-ST-Segment Elevation Acute Coronary Syndrome"[Title/Abstract])) OR ("acute coronary syndrome*"[Title/Abstract])) OR ("Coronary Syndrome*"[Title/Abstract])) OR ("acute cardiac infarction "[Title/Abstract])) OR ("Cardia* infarct*"[Title/Abstract])) OR ("heart infarct*"[Title/Abstract])) OR ("myocardium infarct* "[Title/Abstract])) OR ("Myocardial Infarct*"[Title/Abstract])) OR ("ST elevated MI"[Title/Abstract])) OR ("non ST elevated MI"[Title/Abstract])) OR ("ST elevation MI"[Title/Abstract])) OR ("non ST elevation MI "[Title/Abstract])) OR ("ST segment elevat* myocardial infarction "[Title/Abstract])) OR ("non ST segment elevat* myocardial infarction"[Title/Abstract]))) OR ("ST segment elevation MI"[Title/Abstract])) OR ("non ST segment elevation MI "[Title/Abstract])) OR ("Angina at Rest"[Title/Abstract])) OR ("Cardiovascular Stroke*"[Title/Abstract])) OR ("Heart Attack*"[Title/Abstract]))) AND (("Acute Kidney Injury"[Mesh]) OR (((((((((((((((((((((((((((((((((((((((("kidney injury"[Title/Abstract]) OR ("Renal injury"[Title/Abstract])) OR ("acute kidney failure"[Title/Abstract])) OR ("acute renal failure"[Title/Abstract])) OR ("renal damage"[Title/Abstract])) OR ("kidney damage"[Title/Abstract])) OR ("acute kidney injury"[Title/Abstract])) OR ("acute renal injury"[Title/Abstract])) OR ("AKI"[Title/Abstract])) OR ("CAN"[Title/Abstract])) OR ("radiographic contrast nephropathy"[Title/Abstract])) OR ("contrast induced nephropathy"[Title/Abstract])) OR ("radiocontrast nephropathy"[Title/Abstract])) OR ("CIN"[Title/Abstract])) OR ("contrast associated nephropathy"[Title/Abstract])) OR ("contrast media-induced nephropathy"[Title/Abstract])) OR ("contrast-media induced nephropathy"[Title/Abstract])) OR ("Renal Insufficienc*"[Title/Abstract])) OR ("kidney Insufficienc*"[Title/Abstract])) OR ("Renal Failure*"[Title/Abstract])) OR ("kidney Failure*"[Title/Abstract])) OR ("kidney lesion "[Title/Abstract])) OR ("renal lesion"[Title/Abstract])) OR ("contrast agent induced nephropathy "[Title/Abstract])) OR ("contrast agent induced nephrotoxicity"[Title/Abstract])) OR ("contrast induced acute renal failure"[Title/Abstract])) OR ("contrast induced nephrotoxicity"[Title/Abstract])) OR ("contrast induced nephropathy "[Title/Abstract])) OR ("contrast induced renal dysfunction "[Title/Abstract])) OR ("contrast induced renal failure"[Title/Abstract])) OR ("contrast media induced nephropathy "[Title/Abstract])) OR ("contrast media induced nephrotoxicity"[Title/Abstract])) OR ("contrast media induced renal failure"[Title/Abstract])) OR ("contrast medium induced nephropathy"[Title/Abstract])) OR ("contrast medium induced nephrotoxicity"[Title/Abstract])) OR ("contrast nephropathy "[Title/Abstract])) OR ("contrast nephrotoxicity"[Title/Abstract])) OR ("radio-contrast nephropathy"[Title/Abstract])) OR ("radiocontrast nephropathy "[Title/Abstract])) OR ("radiocontrast-induced nephropathy "[Title/Abstract])))) AND (((((((((("Risk Assessment"[Mesh]) OR ("Risk Factors"[Mesh])) OR ("Regression Analysis"[Mesh])) OR ("Protective Factors"[Mesh])) OR ("Decision Trees"[Mesh])) OR ("Random Forest"[Mesh])) OR ("Neural Networks, Computer"[Mesh])) OR ("Machine Learning"[Mesh])) OR ("Nomograms"[Mesh])) OR ((((((((((((((((((((((((((((((((((((((((((((((((((((((((((((((((((((((((((((((((("Neural Networks, Computer"[Title/Abstract]) OR ("Risk assessment*"[Title/Abstract])) OR ("Risks and Benefits"[Title/Abstract])) OR ("Benefits and Risks"[Title/Abstract])) OR ("Risk Analy*"[Title/Abstract])) OR ("Benefit-risk"[Title/Abstract])) OR ("Benefit risk"[Title/Abstract])) OR ("Risk benefit"[Title/Abstract])) OR ("Risk-benefit"[Title/Abstract])) OR ("Forecast* model*"[Title/Abstract])) OR ("risk prediction score"[Title/Abstract])) OR ("risk adjustment"[Title/Abstract])) OR ("risk evaluation"[Title/Abstract])) OR ("risk factor*"[Title/Abstract])) OR ("Population* at Risk"[Title/Abstract])) OR ("Risk Score*"[Title/Abstract])) OR ("risk prediction model*"[Title/Abstract])) OR ("risk assessment model*"[Title/Abstract])) OR ("Protection"[Title/Abstract])) OR ("Regression Analy*"[Title/Abstract])) OR ("regression model*"[Title/Abstract])) OR ("Regression Diagnostics"[Title/Abstract])) OR ("Statistical Regression*"[Title/Abstract])) OR ("relative risk"[Title/Abstract])) OR ("predictive model*"[Title/Abstract])) OR ("predictive simulation"[Title/Abstract])) OR ("prediction model*"[Title/Abstract])) OR ("prognostic model*"[Title/Abstract])) OR ("Predict* factor*"[Title/Abstract])) OR ("prognostic factor*"[Title/Abstract])) OR ("Predict* variable*"[Title/Abstract])) OR ("Protect* factor*"[Title/Abstract])) OR ("decision tree*"[Title/Abstract])) OR ("Random Forest*"[Title/Abstract])) OR ("random decision forest"[Title/Abstract])) OR ("ann"[Title/Abstract])) OR ("ann analysis"[Title/Abstract])) OR ("ann approach"[Title/Abstract])) OR ("ann method*"[Title/Abstract])) OR ("ann model*"[Title/Abstract])) OR ("ann output"[Title/Abstract])) OR ("ann technique*"[Title/Abstract])) OR ("ann training"[Title/Abstract])) OR ("anns "[Title/Abstract])) OR ("artificial nn"[Title/Abstract])) OR ("artificial nns"[Title/Abstract])) OR ("artificial neural network*"[Title/Abstract])) OR ("computational neural network"[Title/Abstract])) OR ("Support Vector*"[Title/Abstract])) OR ("Vector Machine*"[Title/Abstract])) OR ("Vector Network*"[Title/Abstract])) OR ("Svc"[Title/Abstract])) OR ("svm"[Title/Abstract])) OR ("support vector machine"[Title/Abstract])) OR ("svr"[Title/Abstract])) OR ("support vector classifi*"[Title/Abstract])) OR ("support vector regression"[Title/Abstract])) OR ("machine learning"[Title/Abstract])) OR ("Transfer Learning"[Title/Abstract])) OR ("learning machine*"[Title/Abstract])) OR ("Nomogram"[Title/Abstract])) OR ("safety assessment"[Title/Abstract])) OR ("Health Risk Assessment*"[Title/Abstract])) OR ("Benefit-Risk Assessment*"[Title/Abstract])) OR ("Benefit Risk Assessment"[Title/Abstract])) OR ("Risk-Benefit Assessment*"[Title/Abstract])) OR ("Risk Benefit Assessment"[Title/Abstract])) OR ("Risk Factor Score*"[Title/Abstract])) OR ("Random Forest Classification*"[Title/Abstract])) OR ("random forest algorithm*"[Title/Abstract])) OR ("connectionist network"[Title/Abstract])) OR ("connectionist system"[Title/Abstract])) OR ("neural network*"[Title/Abstract])) OR ("Random Forest Algorithm*"[Title/Abstract])) OR ("Computer Neural"[Title/Abstract])) OR ("Network Model*"[Title/Abstract])) OR ("Computational Neural"[Title/Abstract])) OR ("Perceptron*"[Title/Abstract])) OR ("Connectionist Model*"[Title/Abstract])) OR ("Partin Table*"[Title/Abstract])) OR ("Partin Nomogram*"[Title/Abstract])))) AND ((("Percutaneous Coronary Intervention"[Mesh]) OR ("Angioplasty, Balloon, Coronary"[Mesh])) OR ((((((((((((((((((((((((("Percutaneous Coronary Intervention*"[Title/Abstract]) OR ("Percutaneous Coronary Revascularization*"[Title/Abstract])) OR ("PCI"[Title/Abstract])) OR ("percutaneous transluminal coronary intervention"[Title/Abstract])) OR ("PTAS"[Title/Abstract])) OR ("angioplasty"[Title/Abstract])) OR ("primary percutaneous coronary intervention"[Title/Abstract])) OR ("Percutaneous Transluminal Coronary Angioplasty"[Title/Abstract])) OR ("PTCA"[Title/Abstract])) OR ("Angioplasty, Balloon, Coronary"[Title/Abstract])) OR ("Coronary Intervention*"[Title/Abstract])) OR ("Percutaneous Coronary"[Title/Abstract])) OR ("Coronary Revascularization*"[Title/Abstract])) OR ("Transluminal Coronary Balloon Dilation"[Title/Abstract])) OR ("Balloon Dilation"[Title/Abstract])) OR ("Coronary Artery"[Title/Abstract])) OR ("Angioplast*"[Title/Abstract])) OR ("Coronary Balloon"[Title/Abstract])) OR ("Balloon Angioplast*"[Title/Abstract])) OR ("Transluminal Balloon"[Title/Abstract])) OR ("Percutaneous Transluminal Coronary Angioplasty"[Title/Abstract])) OR ("coronary angioplasty"[Title/Abstract])) OR ("p.t.c.a."[Title/Abstract])) OR ("percutaneous coronary transluminal angioplasty"[Title/Abstract])) OR ("transluminal coronary angioplasty"[Title/Abstract]))) 1,863

#24 Search: (("Percutaneous Coronary Intervention"[Mesh]) OR ("Angioplasty, Balloon, Coronary"[Mesh])) OR ((((((((((((((((((((((((("Percutaneous Coronary Intervention*"[Title/Abstract]) OR ("Percutaneous Coronary Revascularization*"[Title/Abstract])) OR ("PCI"[Title/Abstract])) OR ("percutaneous transluminal coronary intervention"[Title/Abstract])) OR ("PTAS"[Title/Abstract])) OR ("angioplasty"[Title/Abstract])) OR ("primary percutaneous coronary intervention"[Title/Abstract])) OR ("Percutaneous Transluminal Coronary Angioplasty"[Title/Abstract])) OR ("PTCA"[Title/Abstract])) OR ("Angioplasty, Balloon, Coronary"[Title/Abstract])) OR ("Coronary Intervention*"[Title/Abstract])) OR ("Percutaneous Coronary"[Title/Abstract])) OR ("Coronary Revascularization*"[Title/Abstract])) OR ("Transluminal Coronary Balloon Dilation"[Title/Abstract])) OR ("Balloon Dilation"[Title/Abstract])) OR ("Coronary Artery"[Title/Abstract])) OR ("Angioplast*"[Title/Abstract])) OR ("Coronary Balloon"[Title/Abstract])) OR ("Balloon Angioplast*"[Title/Abstract])) OR ("Transluminal Balloon"[Title/Abstract])) OR ("Percutaneous Transluminal Coronary Angioplasty"[Title/Abstract])) OR ("coronary angioplasty"[Title/Abstract])) OR ("p.t.c.a."[Title/Abstract])) OR ("percutaneous coronary transluminal angioplasty"[Title/Abstract])) OR ("transluminal coronary angioplasty"[Title/Abstract]))

#23 Search: (((((((((((((((((((((((("Percutaneous Coronary Intervention*"[Title/Abstract]) OR ("Percutaneous Coronary Revascularization*"[Title/Abstract])) OR ("PCI"[Title/Abstract])) OR ("percutaneous transluminal coronary intervention"[Title/Abstract])) OR ("PTAS"[Title/Abstract])) OR ("angioplasty"[Title/Abstract])) OR ("primary percutaneous coronary intervention"[Title/Abstract])) OR ("Percutaneous Transluminal Coronary Angioplasty"[Title/Abstract])) OR ("PTCA"[Title/Abstract])) OR ("Angioplasty, Balloon, Coronary"[Title/Abstract])) OR ("Coronary Intervention*"[Title/Abstract])) OR ("Percutaneous Coronary"[Title/Abstract])) OR ("Coronary Revascularization*"[Title/Abstract])) OR ("Transluminal Coronary Balloon Dilation"[Title/Abstract])) OR ("Balloon Dilation"[Title/Abstract])) OR ("Coronary Artery"[Title/Abstract])) OR ("Angioplast*"[Title/Abstract])) OR ("Coronary Balloon"[Title/Abstract])) OR ("Balloon Angioplast*"[Title/Abstract])) OR ("Transluminal Balloon"[Title/Abstract])) OR ("Percutaneous Transluminal Coronary Angioplasty"[Title/Abstract])) OR ("coronary angioplasty"[Title/Abstract])) OR ("p.t.c.a."[Title/Abstract])) OR ("percutaneous coronary transluminal angioplasty"[Title/Abstract])) OR ("transluminal coronary angioplasty"[Title/Abstract])

#22 Search: "Angioplasty, Balloon, Coronary"[Mesh] Sort by: Most Recent

#21 Search: "Percutaneous Coronary Intervention"[Mesh] Sort by: Most Recent

#20 Search: ((((((((("Risk Assessment"[Mesh]) OR ("Risk Factors"[Mesh])) OR ("Regression Analysis"[Mesh])) OR ("Protective Factors"[Mesh])) OR ("Decision Trees"[Mesh])) OR ("Random Forest"[Mesh])) OR ("Neural Networks, Computer"[Mesh])) OR ("Machine Learning"[Mesh])) OR ("Nomograms"[Mesh])) OR ((((((((((((((((((((((((((((((((((((((((((((((((((((((((((((((((((((((((((((((((("Neural Networks, Computer"[Title/Abstract]) OR ("Risk assessment*"[Title/Abstract])) OR ("Risks and Benefits"[Title/Abstract])) OR ("Benefits and Risks"[Title/Abstract])) OR ("Risk Analy*"[Title/Abstract])) OR ("Benefit-risk"[Title/Abstract])) OR ("Benefit risk"[Title/Abstract])) OR ("Risk benefit"[Title/Abstract])) OR ("Risk-benefit"[Title/Abstract])) OR ("Forecast* model*"[Title/Abstract])) OR ("risk prediction score"[Title/Abstract])) OR ("risk adjustment"[Title/Abstract])) OR ("risk evaluation"[Title/Abstract])) OR ("risk factor*"[Title/Abstract])) OR ("Population* at Risk"[Title/Abstract])) OR ("Risk Score*"[Title/Abstract])) OR ("risk prediction model*"[Title/Abstract])) OR ("risk assessment model*"[Title/Abstract])) OR ("Protection"[Title/Abstract])) OR ("Regression Analy*"[Title/Abstract])) OR ("regression model*"[Title/Abstract])) OR ("Regression Diagnostics"[Title/Abstract])) OR ("Statistical Regression*"[Title/Abstract])) OR ("relative risk"[Title/Abstract])) OR ("predictive model*"[Title/Abstract])) OR ("predictive simulation"[Title/Abstract])) OR ("prediction model*"[Title/Abstract])) OR ("prognostic model*"[Title/Abstract])) OR ("Predict* factor*"[Title/Abstract])) OR ("prognostic factor*"[Title/Abstract])) OR ("Predict* variable*"[Title/Abstract])) OR ("Protect* factor*"[Title/Abstract])) OR ("decision tree*"[Title/Abstract])) OR ("Random Forest*"[Title/Abstract])) OR ("random decision forest"[Title/Abstract])) OR ("ann"[Title/Abstract])) OR ("ann analysis"[Title/Abstract])) OR ("ann approach"[Title/Abstract])) OR ("ann method*"[Title/Abstract])) OR ("ann model*"[Title/Abstract])) OR ("ann output"[Title/Abstract])) OR ("ann technique*"[Title/Abstract])) OR ("ann training"[Title/Abstract])) OR ("anns "[Title/Abstract])) OR ("artificial nn"[Title/Abstract])) OR ("artificial nns"[Title/Abstract])) OR ("artificial neural network*"[Title/Abstract])) OR ("computational neural network"[Title/Abstract])) OR ("Support Vector*"[Title/Abstract])) OR ("Vector Machine*"[Title/Abstract])) OR ("Vector Network*"[Title/Abstract])) OR ("Svc"[Title/Abstract])) OR ("svm"[Title/Abstract])) OR ("support vector machine"[Title/Abstract])) OR ("svr"[Title/Abstract])) OR ("support vector classifi*"[Title/Abstract])) OR ("support vector regression"[Title/Abstract])) OR ("machine learning"[Title/Abstract])) OR ("Transfer Learning"[Title/Abstract])) OR ("learning machine*"[Title/Abstract])) OR ("Nomogram"[Title/Abstract])) OR ("safety assessment"[Title/Abstract])) OR ("Health Risk Assessment*"[Title/Abstract])) OR ("Benefit-Risk Assessment*"[Title/Abstract])) OR ("Benefit Risk Assessment"[Title/Abstract])) OR ("Risk-Benefit Assessment*"[Title/Abstract])) OR ("Risk Benefit Assessment"[Title/Abstract])) OR ("Risk Factor Score*"[Title/Abstract])) OR ("Random Forest Classification*"[Title/Abstract])) OR ("random forest algorithm*"[Title/Abstract])) OR ("connectionist network"[Title/Abstract])) OR ("connectionist system"[Title/Abstract])) OR ("neural network*"[Title/Abstract])) OR ("Random Forest Algorithm*"[Title/Abstract])) OR ("Computer Neural"[Title/Abstract])) OR ("Network Model*"[Title/Abstract])) OR ("Computational Neural"[Title/Abstract])) OR ("Perceptron*"[Title/Abstract])) OR ("Connectionist Model*"[Title/Abstract])) OR ("Partin Table*"[Title/Abstract])) OR ("Partin Nomogram*"[Title/Abstract]))

#19 Search: (((((((((((((((((((((((((((((((((((((((((((((((((((((((((((((((((((((((((((((((("Neural Networks, Computer"[Title/Abstract]) OR ("Risk assessment*"[Title/Abstract])) OR ("Risks and Benefits"[Title/Abstract])) OR ("Benefits and Risks"[Title/Abstract])) OR ("Risk Analy*"[Title/Abstract])) OR ("Benefit-risk"[Title/Abstract])) OR ("Benefit risk"[Title/Abstract])) OR ("Risk benefit"[Title/Abstract])) OR ("Risk-benefit"[Title/Abstract])) OR ("Forecast* model*"[Title/Abstract])) OR ("risk prediction score"[Title/Abstract])) OR ("risk adjustment"[Title/Abstract])) OR ("risk evaluation"[Title/Abstract])) OR ("risk factor*"[Title/Abstract])) OR ("Population* at Risk"[Title/Abstract])) OR ("Risk Score*"[Title/Abstract])) OR ("risk prediction model*"[Title/Abstract])) OR ("risk assessment model*"[Title/Abstract])) OR ("Protection"[Title/Abstract])) OR ("Regression Analy*"[Title/Abstract])) OR ("regression model*"[Title/Abstract])) OR ("Regression Diagnostics"[Title/Abstract])) OR ("Statistical Regression*"[Title/Abstract])) OR ("relative risk"[Title/Abstract])) OR ("predictive model*"[Title/Abstract])) OR ("predictive simulation"[Title/Abstract])) OR ("prediction model*"[Title/Abstract])) OR ("prognostic model*"[Title/Abstract])) OR ("Predict* factor*"[Title/Abstract])) OR ("prognostic factor*"[Title/Abstract])) OR ("Predict* variable*"[Title/Abstract])) OR ("Protect* factor*"[Title/Abstract])) OR ("decision tree*"[Title/Abstract])) OR ("Random Forest*"[Title/Abstract])) OR ("random decision forest"[Title/Abstract])) OR ("ann"[Title/Abstract])) OR ("ann analysis"[Title/Abstract])) OR ("ann approach"[Title/Abstract])) OR ("ann method*"[Title/Abstract])) OR ("ann model*"[Title/Abstract])) OR ("ann output"[Title/Abstract])) OR ("ann technique*"[Title/Abstract])) OR ("ann training"[Title/Abstract])) OR ("anns "[Title/Abstract])) OR ("artificial nn"[Title/Abstract])) OR ("artificial nns"[Title/Abstract])) OR ("artificial neural network*"[Title/Abstract])) OR ("computational neural network"[Title/Abstract])) OR ("Support Vector*"[Title/Abstract])) OR ("Vector Machine*"[Title/Abstract])) OR ("Vector Network*"[Title/Abstract])) OR ("Svc"[Title/Abstract])) OR ("svm"[Title/Abstract])) OR ("support vector machine"[Title/Abstract])) OR ("svr"[Title/Abstract])) OR ("support vector classifi*"[Title/Abstract])) OR ("support vector regression"[Title/Abstract])) OR ("machine learning"[Title/Abstract])) OR ("Transfer Learning"[Title/Abstract])) OR ("learning machine*"[Title/Abstract])) OR ("Nomogram"[Title/Abstract])) OR ("safety assessment"[Title/Abstract])) OR ("Health Risk Assessment*"[Title/Abstract])) OR ("Benefit-Risk Assessment*"[Title/Abstract])) OR ("Benefit Risk Assessment"[Title/Abstract])) OR ("Risk-Benefit Assessment*"[Title/Abstract])) OR ("Risk Benefit Assessment"[Title/Abstract])) OR ("Risk Factor Score*"[Title/Abstract])) OR ("Random Forest Classification*"[Title/Abstract])) OR ("random forest algorithm*"[Title/Abstract])) OR ("connectionist network"[Title/Abstract])) OR ("connectionist system"[Title/Abstract])) OR ("neural network*"[Title/Abstract])) OR ("Random Forest Algorithm*"[Title/Abstract])) OR ("Computer Neural"[Title/Abstract])) OR ("Network Model*"[Title/Abstract])) OR ("Computational Neural"[Title/Abstract])) OR ("Perceptron*"[Title/Abstract])) OR ("Connectionist Model*"[Title/Abstract])) OR ("Partin Table*"[Title/Abstract])) OR ("Partin Nomogram*"[Title/Abstract])

#18 Search: "Nomograms"[Mesh] Sort by: Most Recent

#17 Search: "Machine Learning"[Mesh] Sort by: Most Recent

#16 Search: "Neural Networks, Computer"[Mesh] Sort by: Most Recent

#15 Search: "Random Forest"[Mesh] Sort by: Most Recent

#14 Search: "Decision Trees"[Mesh] Sort by: Most Recent

#13 Search: "Protective Factors"[Mesh] Sort by: Most Recent

#12 Search: "Regression Analysis"[Mesh] Sort by: Most Recent

#11 Search: "Risk Factors"[Mesh] Sort by: Most Recent

#10 Search: "Risk Assessment"[Mesh] Sort by: Most Recent

#9 Search: ("Acute Kidney Injury"[Mesh]) OR (((((((((((((((((((((((((((((((((((((((("kidney injury"[Title/Abstract]) OR ("Renal injury"[Title/Abstract])) OR ("acute kidney failure"[Title/Abstract])) OR ("acute renal failure"[Title/Abstract])) OR ("renal damage"[Title/Abstract])) OR ("kidney damage"[Title/Abstract])) OR ("acute kidney injury"[Title/Abstract])) OR ("acute renal injury"[Title/Abstract])) OR ("AKI"[Title/Abstract])) OR ("CAN"[Title/Abstract])) OR ("radiographic contrast nephropathy"[Title/Abstract])) OR ("contrast induced nephropathy"[Title/Abstract])) OR ("radiocontrast nephropathy"[Title/Abstract])) OR ("CIN"[Title/Abstract])) OR ("contrast associated nephropathy"[Title/Abstract])) OR ("contrast media-induced nephropathy"[Title/Abstract])) OR ("contrast-media induced nephropathy"[Title/Abstract])) OR ("Renal Insufficienc*"[Title/Abstract])) OR ("kidney Insufficienc*"[Title/Abstract])) OR ("Renal Failure*"[Title/Abstract])) OR ("kidney Failure*"[Title/Abstract])) OR ("kidney lesion "[Title/Abstract])) OR ("renal lesion"[Title/Abstract])) OR ("contrast agent induced nephropathy "[Title/Abstract])) OR ("contrast agent induced nephrotoxicity"[Title/Abstract])) OR ("contrast induced acute renal failure"[Title/Abstract])) OR ("contrast induced nephrotoxicity"[Title/Abstract])) OR ("contrast induced nephropathy "[Title/Abstract])) OR ("contrast induced renal dysfunction "[Title/Abstract])) OR ("contrast induced renal failure"[Title/Abstract])) OR ("contrast media induced nephropathy "[Title/Abstract])) OR ("contrast media induced nephrotoxicity"[Title/Abstract])) OR ("contrast media induced renal failure"[Title/Abstract])) OR ("contrast medium induced nephropathy"[Title/Abstract])) OR ("contrast medium induced nephrotoxicity"[Title/Abstract])) OR ("contrast nephropathy "[Title/Abstract])) OR ("contrast nephrotoxicity"[Title/Abstract])) OR ("radio-contrast nephropathy"[Title/Abstract])) OR ("radiocontrast nephropathy "[Title/Abstract])) OR ("radiocontrast-induced nephropathy "[Title/Abstract]))

#8 Search: ((((((((((((((((((((((((((((((((((((((("kidney injury"[Title/Abstract]) OR ("Renal injury"[Title/Abstract])) OR ("acute kidney failure"[Title/Abstract])) OR ("acute renal failure"[Title/Abstract])) OR ("renal damage"[Title/Abstract])) OR ("kidney damage"[Title/Abstract])) OR ("acute kidney injury"[Title/Abstract])) OR ("acute renal injury"[Title/Abstract])) OR ("AKI"[Title/Abstract])) OR ("CAN"[Title/Abstract])) OR ("radiographic contrast nephropathy"[Title/Abstract])) OR ("contrast induced nephropathy"[Title/Abstract])) OR ("radiocontrast nephropathy"[Title/Abstract])) OR ("CIN"[Title/Abstract])) OR ("contrast associated nephropathy"[Title/Abstract])) OR ("contrast media-induced nephropathy"[Title/Abstract])) OR ("contrast-media induced nephropathy"[Title/Abstract])) OR ("Renal Insufficienc*"[Title/Abstract])) OR ("kidney Insufficienc*"[Title/Abstract])) OR ("Renal Failure*"[Title/Abstract])) OR ("kidney Failure*"[Title/Abstract])) OR ("kidney lesion "[Title/Abstract])) OR ("renal lesion"[Title/Abstract])) OR ("contrast agent induced nephropathy "[Title/Abstract])) OR ("contrast agent induced nephrotoxicity"[Title/Abstract])) OR ("contrast induced acute renal failure"[Title/Abstract])) OR ("contrast induced nephrotoxicity"[Title/Abstract])) OR ("contrast induced nephropathy "[Title/Abstract])) OR ("contrast induced renal dysfunction "[Title/Abstract])) OR ("contrast induced renal failure"[Title/Abstract])) OR ("contrast media induced nephropathy "[Title/Abstract])) OR ("contrast media induced nephrotoxicity"[Title/Abstract])) OR ("contrast media induced renal failure"[Title/Abstract])) OR ("contrast medium induced nephropathy"[Title/Abstract])) OR ("contrast medium induced nephrotoxicity"[Title/Abstract])) OR ("contrast nephropathy "[Title/Abstract])) OR ("contrast nephrotoxicity"[Title/Abstract])) OR ("radio-contrast nephropathy"[Title/Abstract])) OR ("radiocontrast nephropathy "[Title/Abstract])) OR ("radiocontrast-induced nephropathy "[Title/Abstract])

#7 Search: "Acute Kidney Injury"[Mesh] Sort by: Most Recent #6 Search: (((("Acute Coronary Syndrome"[Mesh]) OR ("Angina, Unstable"[Mesh])) OR ("Myocardial Infarction"[Mesh])) OR ("ST Elevation Myocardial Infarction"[Mesh])) OR (((((((((((((((((((((((((((((((((((((((("unstable angina pectoris"[Title/Abstract]) OR ("Angina, Unstable"[Title/Abstract])) OR ("ACS"[Title/Abstract])) OR ("UA"[Title/Abstract])) OR ("UAP"[Title/Abstract])) OR ("unstable angina"[Title/Abstract])) OR ("Acute heart infarction"[Title/Abstract])) OR ("AMI "[Title/Abstract])) OR ("acute myocardial infarction"[Title/Abstract])) OR ("STEMI"[Title/Abstract])) OR ("ST Elevation Myocardial Infarction"[Title/Abstract])) OR ("ST Elevated Myocardial Infarction"[Title/Abstract])) OR ("ST-Segment Elevation Myocardial Infarction"[Title/Abstract])) OR ("STE-ACS"[Title/Abstract])) OR ("ST-Segment Elevation Acute Coronary Syndrome"[Title/Abstract])) OR ("NSTEMI"[Title/Abstract])) OR ("non STEMI"[Title/Abstract])) OR ("Non-ST Elevated Myocardial Infarction"[Title/Abstract])) OR ("Non-ST-Segment Elevation Myocardial Infarction"[Title/Abstract])) OR ("NSTE-ACS"[Title/Abstract])) OR ("Non-ST-Segment Elevation Acute Coronary Syndrome"[Title/Abstract])) OR ("acute coronary syndrome*"[Title/Abstract])) OR ("Coronary Syndrome*"[Title/Abstract])) OR ("acute cardiac infarction "[Title/Abstract])) OR ("Cardia* infarct*"[Title/Abstract])) OR ("heart infarct*"[Title/Abstract])) OR ("myocardium infarct* "[Title/Abstract])) OR ("Myocardial Infarct*"[Title/Abstract])) OR ("ST elevated MI"[Title/Abstract])) OR ("non ST elevated MI"[Title/Abstract])) OR ("ST elevation MI"[Title/Abstract])) OR ("non ST elevation MI "[Title/Abstract])) OR ("ST segment elevat* myocardial infarction "[Title/Abstract])) OR ("non ST segment elevat* myocardial infarction"[Title/Abstract]))) OR ("ST segment elevation MI"[Title/Abstract])) OR ("non ST segment elevation MI "[Title/Abstract])) OR ("Angina at Rest"[Title/Abstract])) OR ("Cardiovascular Stroke*"[Title/Abstract])) OR ("Heart Attack*"[Title/Abstract]))

#5 Search: ((((((((((((((((((((((((((((((((((((((("unstable angina pectoris"[Title/Abstract]) OR ("Angina, Unstable"[Title/Abstract])) OR ("ACS"[Title/Abstract])) OR ("UA"[Title/Abstract])) OR ("UAP"[Title/Abstract])) OR ("unstable angina"[Title/Abstract])) OR ("Acute heart infarction"[Title/Abstract])) OR ("AMI "[Title/Abstract])) OR ("acute myocardial infarction"[Title/Abstract])) OR ("STEMI"[Title/Abstract])) OR ("ST Elevation Myocardial Infarction"[Title/Abstract])) OR ("ST Elevated Myocardial Infarction"[Title/Abstract])) OR ("ST-Segment Elevation Myocardial Infarction"[Title/Abstract])) OR ("STE-ACS"[Title/Abstract])) OR ("ST-Segment Elevation Acute Coronary Syndrome"[Title/Abstract])) OR ("NSTEMI"[Title/Abstract])) OR ("non STEMI"[Title/Abstract])) OR ("Non-ST Elevated Myocardial Infarction"[Title/Abstract])) OR ("Non-ST-Segment Elevation Myocardial Infarction"[Title/Abstract])) OR ("NSTE-ACS"[Title/Abstract])) OR ("Non-ST-Segment Elevation Acute Coronary Syndrome"[Title/Abstract])) OR ("acute coronary syndrome*"[Title/Abstract])) OR ("Coronary Syndrome*"[Title/Abstract])) OR ("acute cardiac infarction "[Title/Abstract])) OR ("Cardia* infarct*"[Title/Abstract])) OR ("heart infarct*"[Title/Abstract])) OR ("myocardium infarct* "[Title/Abstract])) OR ("Myocardial Infarct*"[Title/Abstract])) OR ("ST elevated MI"[Title/Abstract])) OR ("non ST elevated MI"[Title/Abstract])) OR ("ST elevation MI"[Title/Abstract])) OR ("non ST elevation MI "[Title/Abstract])) OR ("ST segment elevat* myocardial infarction "[Title/Abstract])) OR ("non ST segment elevat* myocardial infarction"[Title/Abstract]))) OR ("ST segment elevation MI"[Title/Abstract])) OR ("non ST segment elevation MI "[Title/Abstract])) OR ("Angina at Rest"[Title/Abstract])) OR ("Cardiovascular Stroke*"[Title/Abstract])) OR ("Heart Attack*"[Title/Abstract])

#4 Search: "ST Elevation Myocardial Infarction"[Mesh] Sort by: Most Recent

#3 Search: "Myocardial Infarction"[Mesh] Sort by: Most Recent

#2 Search: "Angina, Unstable"[Mesh] Sort by: Most Recent

#1 Search: "Acute Coronary Syndrome"[Mesh] Sort by: Most Recent

**WOS 3717**

#1 ((((((((((((((((((((((((((((((((((((((((TS=("unstable angina pectoris")) OR TS=("Angina, Unstable")) OR TS=("ACS")) OR TS=("UA")) OR TS=("UAP")) OR TS=("unstable angina")) OR TS=("Acute heart infarction")) OR TS=("AMI")) OR TS=("acute myocardial infarction")) OR TS=("STEMI")) OR TS=("ST Elevation Myocardial Infarction")) OR TS=("ST Elevated Myocardial Infarction")) OR TS=("ST-Segment Elevation Myocardial Infarction")) OR TS=("STE-ACS")) OR TS=("ST-Segment Elevation Acute Coronary Syndrome")) OR TS=("NSTEMI")) OR TS=("non STEMI")) OR TS=("Non-ST Elevated Myocardial Infarction")) OR TS=("Non-ST-Segment Elevation Myocardial Infarction")) OR TS=("NSTE-ACS")) OR TS=("Non-ST-Segment Elevation Acute Coronary Syndrome")) OR TS=("acute coronary syndrome*")) OR TS=("Coronary Syndrome*")) OR TS=("acute cardiac infarction")) OR TS=("Cardia* infarct*")) OR TS=("heart infarct*")) OR TS=("myocardium infarct* ")) OR TS=("Myocardial Infarct*")) OR TS=("ST elevated MI")) OR TS=("non ST elevated MI")) OR TS=("ST elevation MI")) OR TS=("non ST elevation MI")) OR TS=("ST segment elevat* myocardial infarction")) OR TS=("non ST segment elevat* myocardial infarction")) OR TS=("ST segment elevation heart infarction")) OR TS=(non ST segment elevation heart infarction)) OR TS=("ST segment elevation MI")) OR TS=("non ST segment elevation MI")) OR TS=("Angina at Rest")) OR TS=("Cardiovascular Stroke*")) OR TS=("Heart Attack*")

#2 (((((((((((((((((((((((((((((((((((((((((((TS=("kidney injury")) OR TS=("Renal injury")) OR TS=("acute kidney failure")) OR TS=("acute renal failure")) OR TS=("renal damage")) OR TS=("kidney damage")) OR TS=("acute kidney injury")) OR TS=("acute renal injury")) OR TS=("AKI")) OR TS=("CAN")) OR TS=("radiographic contrast nephropathy")) OR TS=("contrast induced nephropathy")) OR TS=("radiocontrast nephropathy")) OR TS=("CIN")) OR TS=("contrast associated nephropathy")) OR TS=("contrast media-induced nephropathy")) OR TS=("contrast-media induced nephropathy")) OR TS=("Renal Insufficienc*")) OR TS=("kidney Insufficienc*")) OR TS=("Renal Failure*")) OR TS=("kidney Failure*")) OR TS=("kidney lesion")) OR TS=("renal lesion")) OR TS=("contrast agent induced nephropathy")) OR TS=("contrast agent induced nephrotoxicity")) OR TS=("contrast induced acute renal failure")) OR TS=("contrast induced nephrotoxicity")) OR TS=("contrast induced nephropathy ")) OR TS=("contrast induced renal dysfunction")) OR TS=("contrast induced renal failure")) OR TS=("contrast media induced nephropathy")) OR TS=("contrast media induced nephrotoxicity")) OR TS=("contrast media induced renal failure")) OR TS=("contrast medium induced nephropathy")) OR TS=("contrast medium induced nephrotoxicity")) OR TS=("contrast medium induced renal failure")) OR TS=("contrast nephropathy")) OR TS=("contrast nephrotoxicity")) OR TS=("contrasting agent-induced nephropathy")) OR TS=("radio-contrast nephropathy")) OR TS=("radio-contrast-induced nephropathy")) OR TS=("radiocontrast nephropathy")) OR TS=("radiocontrast-induced nephropathy")) OR TS=("RC-induced nephropathy")

#3 ((((((((((((((((((((((((((((((((((((((((((((((((((((((((((((((((((((((((((((((((((TS=("Neural Networks, Computer")) OR TS=("Risk assessment*")) OR TS=("Risks and Benefits")) OR TS=("Benefits and Risks")) OR TS=("Risk Analy*")) OR TS=("Benefit-risk")) OR TS=("Benefit risk")) OR TS=("Risk benefit")) OR TS=("Risk-benefit")) OR TS=("Forecast* model*")) OR TS=("risk prediction score")) OR TS=("risk adjustment")) OR TS=("risk evaluation")) OR TS=("risk factor*")) OR TS=("Population* at Risk")) OR TS=("Risk Score*")) OR TS=("risk prediction model*")) OR TS=("risk assessment model*")) OR TS=("Protection")) OR TS=("Regression Analy*")) OR TS=("sregression model analysis")) OR TS=("regression model*")) OR TS=("Regression Diagnostics")) OR TS=("Statistical Regression*")) OR TS=("relative risk")) OR TS=("predictive model*")) OR TS=("predictive simulation")) OR TS=("prediction model*")) OR TS=("prognostic model*")) OR TS=("Predict* factor*")) OR TS=("prognostic factor*")) OR TS=("Predict* variable*")) OR TS=("Protect* factor*")) OR TS=("decision tree*")) OR TS=("Random Forest*")) OR TS=("random decision forest")) OR TS=("ann")) OR TS=("ann analysis")) OR TS=("ann approach")) OR TS=("ann method*")) OR TS=("ann model*")) OR TS=("ann output")) OR TS=("ann technique*")) OR TS=("ann training")) OR TS=("anns")) OR TS=("algorithmic neural network")) OR TS=("artificial nn")) OR TS=("artificial nns")) OR TS=("artificial neural network*")) OR TS=("computational neural network")) OR TS=("Support Vector*")) OR TS=("Vector Machine*")) OR TS=("Vector Network*")) OR TS=("Svc")) OR TS=("svm")) OR TS=("support vector machine")) OR TS=("svr")) OR TS=("support vector classifi*")) OR TS=("support vector regression")) OR TS=("machine learning")) OR TS=("Transfer Learning")) OR TS=("learning machine*")) OR TS=("Nomogram")) OR TS=("safety assessment")) OR TS=("Health Risk Assessment*")) OR TS=("Benefit-Risk Assessment*")) OR TS=("Benefit Risk Assessment")) OR TS=("Risk-Benefit Assessment*")) OR TS=("Risk Benefit Assessment")) OR TS=("Risk Factor Score*")) OR TS=("Random Forest Classification*")) OR TS=("random forest algorithm*")) OR TS=("connectionist network")) OR TS=("connectionist system")) OR TS=("neural network*")) OR TS=("Random Forest Algorithm*")) OR TS=("Computer Neural")) OR TS=("Network Model*")) OR TS=("Computational Neural")) OR TS=("Perceptron*")) OR TS=("Connectionist Model*")) OR TS=("Partin Table*")) OR TS=("Partin Nomogram*")

#4 (((((((((((((((((((((((((TS=("Percutaneous Coronary Intervention*")) OR TS=("Percutaneous Coronary Revascularization*")) OR TS=("PCI")) OR TS=("percutaneous transluminal coronary intervention")) OR TS=("percutaneous endoluminal stenting")) OR TS=("PTAS")) OR TS=("angioplasty")) OR TS=("primary percutaneous coronary intervention")) OR TS=("Percutaneous Transluminal Coronary Angioplasty")) OR TS=("PTCA")) OR TS=("Angioplasty, Balloon, Coronary")) OR TS=("Coronary Intervention*")) OR TS=("Percutaneous Coronary")) OR TS=("Coronary Revascularization*")) OR TS=("Transluminal Coronary Balloon Dilation")) OR TS=("Balloon Dilation")) OR TS=("Coronary Artery")) OR TS=("Angioplast*")) OR TS=("Coronary Balloon")) OR TS=("Balloon Angioplast*")) OR TS=("Transluminal Balloon")) OR TS=("Percutaneous Transluminal Coronary Angioplasty")) OR TS=("coronary angioplasty")) OR TS=("p.t.c.a.")) OR TS=("percutaneous coronary transluminal angioplasty")) OR TS=("transluminal coronary angioplasty")

#5 #1 AND #2 AND #3 AND #4

**Cochrane library 2848**

#1 MeSH descriptor: [Acute Coronary Syndrome] explode all trees

#2 MeSH descriptor: [Angina, Unstable] explode all trees

#3 MeSH descriptor: [Myocardial Infarction] explode all trees

#4 MeSH descriptor: [ST Elevation Myocardial Infarction] explode all trees

#5 MeSH descriptor: [Non-ST Elevated Myocardial Infarction] explode all trees

#6 (acute coronary syndrome):ti,ab,kw OR (unstable angina pectoris):ti,ab,kw OR (Angina, Unstable):ti,ab,kw OR (heart infarction):ti,ab,kw OR (Myocardial Infarction):ti,ab,kw OR (ACS):ti,ab,kw OR (UA):ti,ab,kw OR (UAP):ti,ab,kw OR (unstable angina):ti,ab,kw OR (Acute heart infarction):ti,ab,kw OR (AMI):ti,ab,kw OR (acute myocardial infarction):ti,ab,kw OR (STEMI):ti,ab,kw OR (ST Elevation Myocardial Infarction):ti,ab,kw OR (ST Elevated Myocardial Infarction):ti,ab,kw OR (ST-Segment Elevation Myocardial Infarction):ti,ab,kw OR (STE-ACS):ti,ab,kw OR (ST-Segment Elevation Acute Coronary Syndrome):ti,ab,kw OR (NSTEMI):ti,ab,kw OR (non STEMI):ti,ab,kw OR (Non-ST Elevated Myocardial Infarction):ti,ab,kw OR (Non-ST-Segment Elevation Myocardial Infarction):ti,ab,kw OR (NSTE-ACS):ti,ab,kw OR (Non-ST-Segment Elevation Acute Coronary Syndrome):ti,ab,kw OR (acute coronary syndrome*):ti,ab,kw OR (Coronary Syndrome*):ti,ab,kw OR (acute cardiac infarction):ti,ab,kw OR (Cardia* infarct*):ti,ab,kw OR (heart infarct*):ti,ab,kw OR (myocardium infarct* ):ti,ab,kw OR (Myocardial Infarct*):ti,ab,kw OR (ST elevated MI):ti,ab,kw OR (non ST elevated MI):ti,ab,kw OR (ST elevation MI):ti,ab,kw OR (non ST elevation MI):ti,ab,kw OR (ST segment elevat* myocardial infarction):ti,ab,kw OR (non ST segment elevat* myocardial infarction):ti,ab,kw OR (ST segment elevation heart infarction):ti,ab,kw OR (non ST segment elevation heart infarction):ti,ab,kw OR (ST segment elevation MI):ti,ab,kw OR (non ST segment elevation MI):ti,ab,kw OR (Angina at Rest):ti,ab,kw OR (Cardiovascular Stroke*):ti,ab,kw OR (Heart Attack*):ti,ab,kw

#7 #1 OR #2 OR #3 OR #4 OR #5 OR #6

#8 MeSH descriptor: [Acute Kidney Injury] explode all trees

#9 (kidney injury):ti,ab,kw OR (Renal injury):ti,ab,kw OR (acute kidney failure):ti,ab,kw OR (acute renal failure):ti,ab,kw OR (renal damage):ti,ab,kw OR (kidney damage):ti,ab,kw OR (acute kidney injury):ti,ab,kw OR (acute renal injury):ti,ab,kw OR (AKI):ti,ab,kw OR (CAN):ti,ab,kw OR (radiographic contrast nephropathy):ti,ab,kw OR (contrast induced nephropathy):ti,ab,kw OR (radiocontrast nephropathy):ti,ab,kw OR (CIN):ti,ab,kw OR (contrast associated nephropathy):ti,ab,kw OR (contrast media-induced nephropathy):ti,ab,kw OR (contrast-media induced nephropathy):ti,ab,kw OR (Renal Insufficienc*):ti,ab,kw OR (kidney Insufficienc*):ti,ab,kw OR (Renal Failure*):ti,ab,kw OR (kidney Failure*):ti,ab,kw OR (kidney lesion):ti,ab,kw OR (renal lesion):ti,ab,kw OR (contrast agent induced nephropathy):ti,ab,kw OR (contrast agent induced nephrotoxicity):ti,ab,kw OR (contrast induced acute renal failure):ti,ab,kw OR (contrast induced nephrotoxicity):ti,ab,kw OR (contrast induced nephropathy ):ti,ab,kw OR (contrast induced renal dysfunction):ti,ab,kw OR (contrast induced renal failure):ti,ab,kw OR (contrast media induced nephropathy):ti,ab,kw OR (contrast media induced nephrotoxicity):ti,ab,kw OR (contrast media induced renal failure):ti,ab,kw OR (contrast medium induced nephropathy):ti,ab,kw OR (contrast medium induced nephrotoxicity):ti,ab,kw OR (contrast medium induced renal failure):ti,ab,kw OR (contrast nephropathy):ti,ab,kw OR (contrast nephrotoxicity):ti,ab,kw OR (contrasting agent-induced nephropathy):ti,ab,kw OR (radio-contrast nephropathy):ti,ab,kw OR (radio-contrast-induced nephropathy):ti,ab,kw OR (radiocontrast nephropathy):ti,ab,kw OR (radiocontrast-induced nephropathy):ti,ab,kw OR (RC-induced nephropathy):ti,ab,kw

#10 #8 OR #9

#11 MeSH descriptor: [Risk Assessment] explode all trees

#12 MeSH descriptor: [Risk Factors] explode all trees

#13 MeSH descriptor: [Regression Analysis] explode all trees

#14 MeSH descriptor: [Protective Factors] explode all trees

#15 MeSH descriptor: [Decision Trees] explode all trees

#16 MeSH descriptor: [Random Forest] explode all trees

#17 MeSH descriptor: [Neural Networks, Computer] explode all trees

#18 MeSH descriptor: [Machine Learning] explode all trees

#19 MeSH descriptor: [Nomograms] explode all trees

#20 (Neural Networks, Computer ):ti,ab,kw OR (Risk assessment*):ti,ab,kw OR (Risks and Benefits):ti,ab,kw OR (Benefits and Risks):ti,ab,kw OR (Risk Analy*):ti,ab,kw OR (Benefit-risk):ti,ab,kw OR (Benefit risk):ti,ab,kw OR (Risk benefit):ti,ab,kw OR (Risk-benefit):ti,ab,kw OR (Forecast* model*):ti,ab,kw OR (risk prediction score):ti,ab,kw OR (risk adjustment):ti,ab,kw OR (risk evaluation):ti,ab,kw OR (risk factor*):ti,ab,kw OR (Population* at Risk):ti,ab,kw OR (Risk Score*):ti,ab,kw OR (risk prediction model*):ti,ab,kw OR (risk assessment model*):ti,ab,kw OR (Protection):ti,ab,kw OR (Regression Analy*):ti,ab,kw OR (sregression model analysis):ti,ab,kw OR (regression model*):ti,ab,kw OR (Regression Diagnostics):ti,ab,kw OR (Statistical Regression*):ti,ab,kw OR (relative risk):ti,ab,kw OR (predictive model*):ti,ab,kw OR (predictive simulation):ti,ab,kw OR (prediction model*):ti,ab,kw OR (prognostic model*):ti,ab,kw OR (Predict* factor*):ti,ab,kw OR (prognostic factor*):ti,ab,kw OR (Predict* variable*):ti,ab,kw OR (Protect* factor*):ti,ab,kw OR (decision tree*):ti,ab,kw OR (Random Forest*):ti,ab,kw OR (random decision forest):ti,ab,kw OR (ann):ti,ab,kw OR (ann analysis):ti,ab,kw OR (ann approach):ti,ab,kw OR (ann method*):ti,ab,kw OR (ann model*):ti,ab,kw OR (ann output):ti,ab,kw OR (ann technique*):ti,ab,kw OR (ann training):ti,ab,kw OR (anns):ti,ab,kw OR (algorithmic neural network):ti,ab,kw OR (artificial nn):ti,ab,kw OR (artificial nns):ti,ab,kw OR (artificial neural network*):ti,ab,kw OR (computational neural network):ti,ab,kw OR (Support Vector*):ti,ab,kw OR (Vector Machine*):ti,ab,kw OR (Vector Network*):ti,ab,kw OR (Svc):ti,ab,kw OR (svm):ti,ab,kw OR (support vector machine):ti,ab,kw OR (svr):ti,ab,kw OR (support vector classifi*):ti,ab,kw OR (support vector regression):ti,ab,kw OR (machine learning):ti,ab,kw OR (Transfer Learning):ti,ab,kw OR (learning machine*):ti,ab,kw OR (Nomogram):ti,ab,kw OR (safety assessment):ti,ab,kw OR (Health Risk Assessment*):ti,ab,kw OR (Benefit-Risk Assessment*):ti,ab,kw OR (Benefit Risk Assessment):ti,ab,kw OR (Risk-Benefit Assessment*):ti,ab,kw OR (Risk Benefit Assessment):ti,ab,kw OR (Risk Factor Score*):ti,ab,kw OR (Random Forest Classification*):ti,ab,kw OR (random forest algorithm*):ti,ab,kw OR (connectionist network):ti,ab,kw OR (connectionist system):ti,ab,kw OR (neural network*):ti,ab,kw OR (Random Forest Algorithm*):ti,ab,kw OR (Computer Neural):ti,ab,kw OR (Network Model*):ti,ab,kw OR (Computational Neural):ti,ab,kw OR (Perceptron*):ti,ab,kw OR (Connectionist Model*):ti,ab,kw OR (Partin Table*):ti,ab,kw OR (Partin Nomogram*):ti,ab,kw

#21 #11 OR #12 OR #13 OR #14 OR #15 OR #16 OR #17 OR #18 OR #19 OR #20

#22 MeSH descriptor: [Percutaneous Coronary Intervention] explode all trees

#23 MeSH descriptor: [Angioplasty, Balloon, Coronary] explode all trees

#24 (Percutaneous Coronary Intervention*):ti,ab,kw OR (Percutaneous Coronary Revascularization*):ti,ab,kw OR (PCI):ti,ab,kw OR (percutaneous transluminal coronary intervention):ti,ab,kw OR (percutaneous endoluminal stenting):ti,ab,kw OR (PTAS):ti,ab,kw OR (angioplasty):ti,ab,kw OR (primary percutaneous coronary intervention):ti,ab,kw OR (Percutaneous Transluminal Coronary Angioplasty):ti,ab,kw OR (PTCA):ti,ab,kw OR (Angioplasty, Balloon, Coronary):ti,ab,kw OR (Coronary Intervention*):ti,ab,kw OR (Percutaneous Coronary):ti,ab,kw OR (Coronary Revascularization*):ti,ab,kw OR (Transluminal Coronary Balloon Dilation):ti,ab,kw OR (Balloon Dilation):ti,ab,kw OR (Coronary Artery):ti,ab,kw OR (Angioplast*):ti,ab,kw OR (Coronary Balloon):ti,ab,kw OR (Balloon Angioplast*):ti,ab,kw OR (Transluminal Balloon):ti,ab,kw OR (Percutaneous Transluminal Coronary Angioplasty):ti,ab,kw OR (coronary angioplasty):ti,ab,kw OR (p.t.c.a.):ti,ab,kw OR (percutaneous coronary transluminal angioplasty):ti,ab,kw OR (transluminal coronary angioplasty):ti,ab,kw

#25 #22 OR #23 OR #24

#26 #7 AND #10 AND #21 AND #25

# Supplementary Figures and Tables

## Supplementary Figures

**
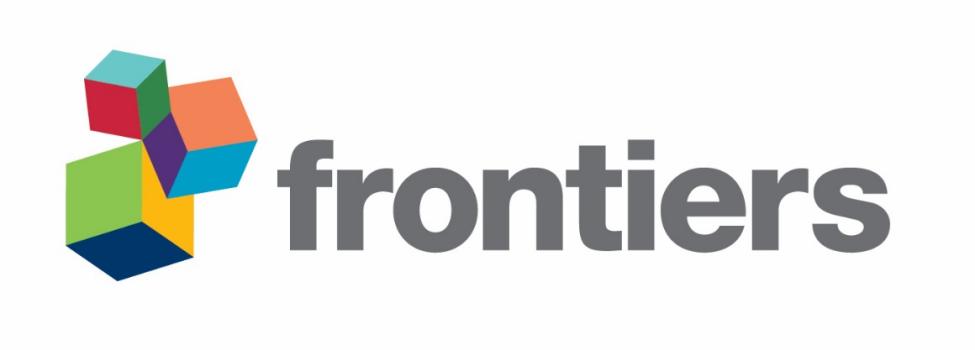
**

**Supplementary Figure S1.**

Supplemental Figure S1 Sensitivity analysis of derivation models

**Supplementary Figure S2.**

Supplemental Figure S2 funnel plots of derivation models

**Supplementary Figure S3.**

Supplemental Figure S3 Sensitivity analysis of validation models

**Supplementary Figure S4.**

Supplemental Figure S4 funnel plots of validation models

## Supplementary Table

Supplemental Table S1 Overview of hydration and contrast agent usage
